# Plant‐Derived Viral Nanoparticles Enable Simultaneous Guidance of Neuronal Cell Outgrowth and Targeting of Neurodifferentiation Pathways

**DOI:** 10.1002/smll.202509395

**Published:** 2025-11-21

**Authors:** Mira Ritter, Natalija Stojanović, Simon Zschieschang, Johannes Grader, MHD Naeem Assasa, Eva Miriam Buhl, Andrea Coschiera, Stefan Schillberg, Juliane Schuphan, Horst Fischer

**Affiliations:** ^1^ Department of Dental Materials and Biomaterials Research RWTH Aachen University Hospital Pauwelsstraße 30 52074 Aachen Germany; ^2^ Institute for Molecular Biotechnology RWTH Aachen University Worringerweg 1 52074 Aachen Germany; ^3^ Electron Microscopy Facility Institute of Pathology RWTH Aachen University Hospital Pauwelsstrasse 30 52074 Aachen Germany; ^4^ Fraunhofer Institute for Molecular Biology and Applied Ecology IME Forckenbeckstraße 6 52074 Aachen Germany

**Keywords:** growth guidance, neurodifferentiation, plant virus nanoparticles, potato virus X, tobacco mosaic virus, VNP‐cell interaction

## Abstract

Differentiating neuronal cells *in vitro* is a complex process that can be significantly enhanced by using a combination of functional peptides and nanostructured scaffolds in combination. However, applying these elements simultaneously remains challenging. Here, a novel neural tissue engineering approach that uses plant‐derived viral nanoparticles (VNPs) to simultaneously promote neuronal differentiation and growth guidance is presented. It is hypothesized that the simultaneous alignment and promotion of neurodifferentiation could be achieved by using genetically engineered potato virus X and tobacco mosaic virus, which display high local concentrations of functional peptides derived from laminin (RGD and IKVAV) and brain‐derived neurotrophic factor. Immunostaining, gene analysis, immunoprecipitation, and western blotting are employed to evaluate the effect of VNPs on neurodifferentiation and their mechanism of action via cell membrane receptors. 3D printing with sacrificial materials is used to align the VNPs, as confirmed by scanning electron microscopy. This approach creates an orientated microarchitecture that simultaneously combines growth guidance and pathway targeting. The incorporation of growth‐factor‐like peptides onto the VNP surface through genetic engineering represents a significant advancement in this area of research. This provides unparalleled control over neural cell differentiation and neurite outgrowth by utilizing plant‐derived, bioactive, and biomimetic nanoparticles as a multifunctional scaffold base.

## Graphical abstract

A neuronal tissue engineering approach employing plant‐derived viral nanoparticles (VNPs) that have been genetically engineered to display functional peptides is presented. These VNPs simultaneously promote neuronal differentiation and directional growth. Aligning VNPs creates a defined microarchitecture that guides neurite extension and enhances the differentiation of neural precursor cells.

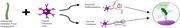

## 1 Introduction

Innervation, alongside angiogenesis, is especially important for restoring the functionality of damaged tissues, which underscores the important role of neural tissue engineering.^[^ ^1^ ^]^ Spatiotemporal guidance of neural systems is crucial for adequately mimicking native organizational processes, enhancing 3D tissue regeneration and functionalization, and ensuring long‐term stability.^[^ ^2^, ^3^ ^]^ Furthermore, neuronal differentiation and the formation of complex neuronal networks depend heavily upon the microarchitecture of the surrounding material.^[^ ^3^, ^4^ ^]^ In vivo, this material is the extracellular matrix (ECM), which provides structural support and bioactive domains that regulate cellular attachment and behavior.^[^ ^5^, ^6^ ^]^ These bioactive domains are also used *in vitro* to promote cell attachment and differentiation.^[^ ^7^, ^8^ ^]^ Examples of these functional peptides include IKVAV and RGD found on laminin α‐chain, and YIGSR on laminin β‐chain.^[^ ^9^ ^]^ Apart from providing cell receptor binding motifs, ECM substrates must provide specific microscale architecture to evoke an appropriate cellular response and support neural tissue function.^[^ ^10^, ^11^, ^12^ ^]^ Additionally, neuronal differentiation both in vivo and in vitro depends heavily upon growth factors such as the brain‐derived neurotrophic factor (BDNF), which controls neuronal development, neuroprotection, and the modulation of synaptic interactions.^[^ ^10^, ^13^, ^14^ ^]^ We hypothesize that both the requirements of providing bioactive domains as well as forming microarchitecture to guide the cells can be fulfilled by plant virus nanoparticles (VNPs). Molecular farming in plants can produce large quantities of VNPs, and numerous copies of one or more identical coat protein (CP) subunits self‐assemble into defined spherical or rod‐shaped particles.^[^ ^15^ ^]^ Engineered plant VNPs such as potato virus X (PVX) and tobacco mosaic virus (TMV) stand out due to their enhanced cytocompatibility and remarkably high local concentration of functional peptides on the viral surface due to their high‐aspect ratio.^[^ ^5^, ^16^ ^]^ VNPs form filamentous fibril and network‐like structures that provide a biomimetic ECM‐like microenvironment. Genetically engineered plant VNPs that present cell‐adhesion motifs on their surfaces have been successfully incorporated into hydrogels for bone tissue substitutes.^[^ ^15^, ^17^, ^18^, ^19^ ^]^

PVX comprises 1270 CP subunits that assemble around a 6.5‐kb genomic single‐stranded RNA to form flexuous, rod‐shaped VNPs. The N‐terminus of the PVX CP is exposed on the viral surface, enabling the attachment of functional peptides.^[^ ^5^ ^]^ In contrast, the well‐characterized, rod‐shaped TMV consists of 2130 identical CP copies and has been extensively used for various applications, including vaccine development, stem cell differentiation, bioimaging, and drug delivery.^[^ ^20^ ^]^ Both the N‐ and C‐termini of the TMV CP are accessible on the viral surface. However, genetic fusions at the 3′ terminus demonstrate greater tolerance for inserted sequences intended for surface display.^[^ ^16^ ^]^ Previous studies have shown that TMV VNPs promote neurite outgrowth in the mouse neural crest‐derived cell line N2a and can be orientated by shear stress within capillaries.^[^ ^16^ ^]^ These aligned VNPs were found to dictate the directional neurite outgrowth of the N2A cells. As such, they are a promising approach for neural tissue engineering.^[^ ^16^ ^]^ Apart from TMV VNPs^[^ ^16^, ^21^ ^]^ and M13 bacteriophages^[^ ^22^ ^]^ application of similar plant VNPs for neuro‐differentiation has not been extensively investigated.

In the present study, we synthesized five modified versions of two plant VNPs that have different structural characteristics: PVX‐RGD, PVX‐IKVAV, PVX‐BDNF‐2A, TMV‐RGD, and TMV‐IKVAV. These modifications were achieved through genetic engineering and produced via molecular farming. We hypothesized that these functionalized and aligned VNPs could target neurodifferentiation pathways and guide neuronal cell outgrowth simultaneously. To evaluate the effects of these VNPs on the neurodifferentiation of SH‐SY5Y neuroblastoma cells, we employed various methodologies including the assessment of the interaction mechanism, pathway activation downstream of TrkB and Itgβ1, gene expression analysis, and immunostaining with image analysis. Additionally, we used scanning electron microscopy to examine particle orientation and immunostaining to visualize the alignment of neurons in relation to the VNPs.

## 2 Results

### 2.1 Production and Characterization of Plant Viral Nanoparticles

**Figure 1:**
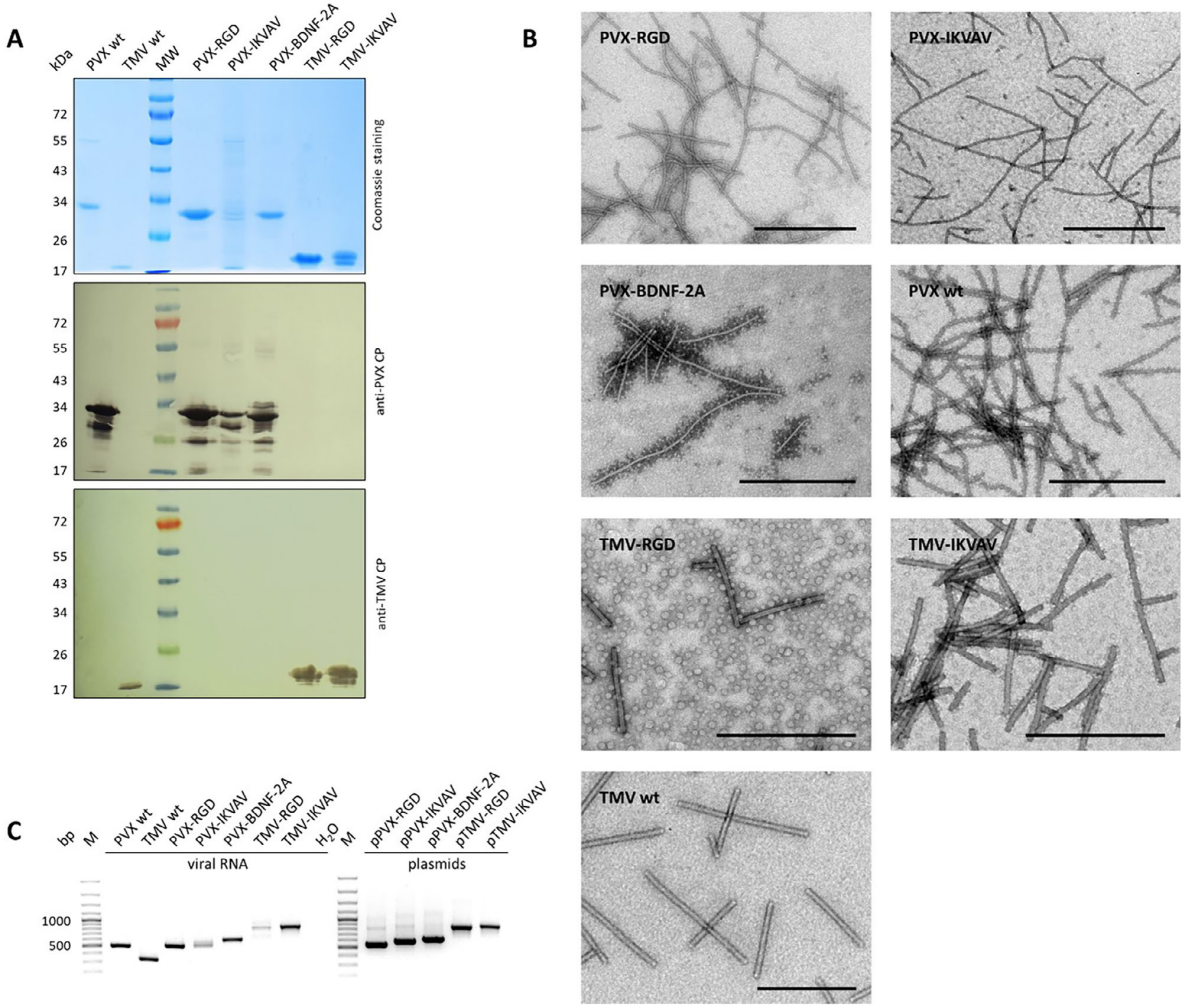
Analysis of plant virus nanoparticles displaying functional motifs. A) Purified PVX or TMV VNPs displaying RGD, IKVAV, or BDNF were separated by SDS‐PAGE, and proteins were detected with Coomassie Brilliant Blue or by western blot using anti‐PVX CP or anti‐TMV CP/GAR^AP^ antibodies to detect the ≈30‐kDa modified PVX CP or the ≈20‐kDa TMV CP. B) Electron micrographs of VNPs. Scale bar represents 500 nm. C) Analysis of encapsidated viral RNA.

VNPs were modified to enhance their functional properties by incorporating bioactive peptides that facilitate targeted interactions with neuronal cells. The IKVAV, RGD, or BDNF coding sequences were genetically fused to either the 5′ or 3′ end of the PVX or TMV *CP* genes, resulting in the vectors pPVX‐IKVAV, pPVX‐BDNF‐2A, and pTMV‐IKVAV. It is important to highlight that pPVX‐BDNF‐2A uses a foot‐and‐mouth disease virus (FMDV 2A) ribosomal skipping sequence. This sequence has been shown to facilitate the assembly process of CPs carrying unfavorable fusions.^[^ ^5^ ^]^ In these cases, ≈25% of the CP subunits contain the functional peptide.^[^ ^23^ ^]^ These viral vectors, along with those developed in previous studies (pPVX‐RGD^[^ ^15^ ^]^ and pTMV‐RGD^[^ ^18^ ^]^), were used to infect *Nicotiana benthamiana* plants, resulting in the systemic production of VNPs. After purification, SDS‐PAGE and western blot analysis using anti‐PVX CP or anti‐TMV CP primary antibodies confirmed the correct molecular weights of the modified PVX CPs (≈30 kDa) and TMV CPs (≈20 kDa) (**Figure** **1**A). The VNPs were then adsorbed onto grids for transmission electron microscopy (Figure 1B) to verify their structural integrity. The modified PVX and TMV VNPs that display RGD, IKVAV, or BDNF showed structural similarities to their wild‐type counterparts. Specifically, PVX maintained its characteristic flexible rod shape, measuring 15 × 515 nm, and TMV retained its rigid rod structure measuring 18 × 300 nm. These results indicate that the modifications did not adversely affect the overall morphology of the VNPs. The encapsidated viral RNA was analyzed using RT‐PCR to synthesize cDNA and amplify the genetic fusions (Figure 1C). The amplified fragments were sequenced, and the successful incorporations of the RGD, IKVAV, or BDNF modifications without point mutations were confirmed. This ensures the functionality of the VNPs for further cell applications.

### 2.2 Interactions of Plant Viral Nanoparticles and Cells

**Figure 2:**
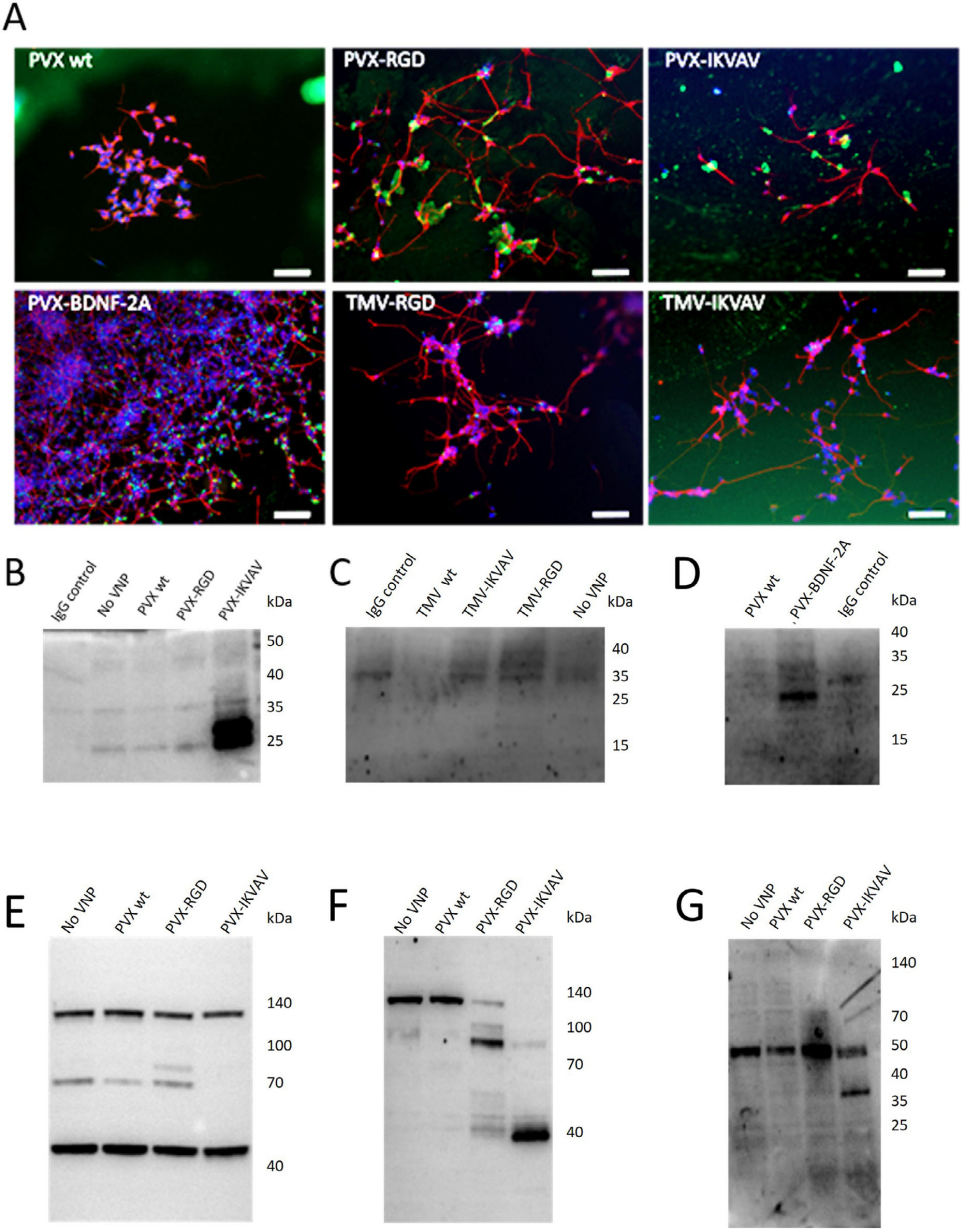
Interactions of VNPs and cells. A) Immunofluorescence staining of differentiated neurons with β‐3 tubulin (red), DAPI‐labeled cell nuclei (blue), and VNPs (green). Scale bar represents 100 µm. B–D) Investigation of cell membrane receptor–VNP complexes after incubation of neuroblastoma cells with VNPs. Membrane‐chemiluminescence overlay of immunoprecipitated samples is shown post western blotting and antibody incubation. B) Immunoprecipitated integrin β1 with PVX VNPs displaying IKVAV or RGD. C) Immunoprecipitated integrin β1 with TMV VNPs displaying IKVAV or RGD. D) Immunoprecipitated pan‐Trk receptor with PVX displaying BDNF‐derived peptide. E–G) Investigation of focal adhesion kinase (FAK) phosphorylation, post 30‐minute incubation with PVX VNPs displaying laminin‐derived peptides. Membrane, chemiluminescence, and overlay are shown post western blotting and antibody incubation. E) Total FAK; F) p‐FAK (Tyr397); G) p‐FAK (Tyr925).

The initial investigations of cell–VNP interactions used immunofluorescent microscopy to focus on the localization of VNP and cell signals. This technique revealed different levels of interaction between β‐3 tubulin‐positive neuronal cells and VNPs (**Figure** **2**A). In the PVX wild‐type (wt) group, no green signal was present in contact with neurons. In contrast, the PVX‐RGD and PVX‐IKVAV VNPs, visible as green dots, came into contact with the cell body or neurites. Some interaction of the VNPs with the neurites was noticed in the TMV‐RGD and TMV‐IKVAV groups. However, these interactions were mostly localized at the cell body and not at the neurites. Furthermore, there was less cell‐VNP interaction compared to the PVX equivalents. In the PVX‐BDNF‐2A group, the green signal derived from PVX‐BDNF‐2A was found along the entire neuron (Figure S2, Supporting Information).

Further experiments focused on revealing the specific interaction mechanism between VNPs and cell receptors. VNPs carrying RGD and IKVAV were tested for interaction with the integrin β1 receptor (Figure 2B,C), and the presence of PVX‐BDNF‐2A was investigated in the immunoprecipitated pan‐Trk receptor (Figure 2D). A strong chemiluminescent signal at ≈25 kDa was present in the PVX‐IKVAV group, confirming the IKVAV peptide's interaction with integrin β1 (Figure 2B). In the PVX wt and PVX‐RGD groups, the presence of the VNPs could not be confirmed as the signal intensity is comparable to that of the IgG light chain. A strong signal was also present at ≈30 kDa in the PVX‐BDNF‐2A group (Figure 2D), confirming the presence of PVX‐BDNF‐2A in the immunoprecipitated Trk receptor complex. The absence of a signal from PVX wt indicates that interaction with Trk occurs specifically through the BDNF peptide present on the PVX‐BDNF‐2A VNPs. In the groups where cells were incubated with TMV VNPs and then had their integrin β1 receptor pulled down, no signal corresponding to TMV VNPs at ≈20 kDa was detected (Figure 2C). This indicates that these VNPs did not interact with the cells through the integrin β1 receptor.

Downstream activation of integrin receptors was investigated through focal adhesion kinase (FAK) using western blotting (Figure 2E–G). Imaging of the membranes revealed differences in the phosphorylation of FAK isomers among the various PVX groups. These findings suggest that the cellular effects of the PVX nanoparticles are due to the peptide displayed by the PVX VNP. A comparison of the molecular weight of the FAK bands between the negative control (No VNP) and PVX wt groups revealed no significant difference. However, the primary focus of the investigation revealed that in the PVX‐RGD and PVX‐IKVAV groups shorter FAK isomers appeared to be primarily phosphorylated. pFAK (Tyr397) showed strong bands at ≈140 kDa for No VNP and PVX wt, while PVX‐RGD and PVX‐IKVAV showed weaker and no bands, respectively, at this molecular weight. In the PVX‐RGD group, however, there was a strong band at ≈100 kDa and another pair of bands at ≈40 kDa. The PVX‐IKVAV group also had a relatively weak band at ≈100 kDa and the strongest band at ≈40 kDa. When stained with pFAK (Tyr925), the PVX‐IKVAV group differs from the others due to the presence of a strong band at ≈40 kDa. Confirmation of different FAK isomers of different molecular weights was done at the mRNA level (Section S2, Supporting Information). The results are shown in Figure S1 (Supporting Information).

### 2.3 Influence of VNPs on Neuronal Differentiation

**Figure 3:**
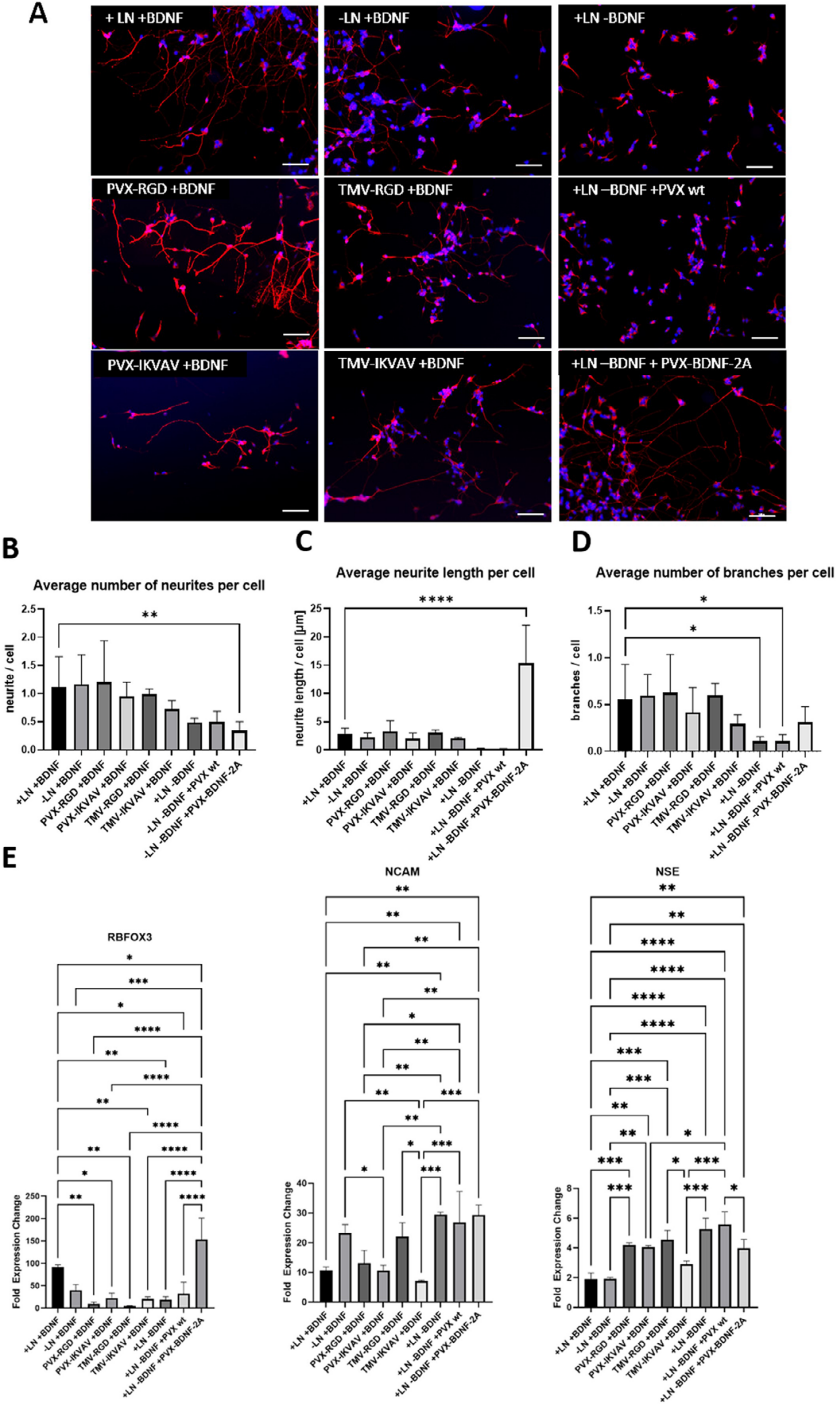
Analysis of neurodifferentiation of SH‐SY5Y cells after 22 days. A) Immunostaining of cells with β‐3 tubulin (red) and DAPI‐labeled cell nuclei (blue). Scale bar represents 100 µm. B) Analysis of neuron morphology (average number of neurites; average neurite length; average number of branches); *n* = 6. C) qPCR analysis of fold expression changes of neuronal markers RNA binding fox‐1 homolog 3 (RBFOX3), Neuronal Cell Adhesion Molecule 1 (NCAM) and Neuron Specific Enolase (NSE); *n* = 3, *p* ≤ 0.05 (*), *p* ≤ 0.001 (**), *p* ≤ 0.0001 (***), *p* ≤ 0.00001 (****), statistical analysis: one‐way analysis of variance.

The extent of SH‐SY5Y neurodifferentiation after 22 days can be predicted by analyzing the expression of neuronal markers, cell morphology, and qPCR.^[^ ^24^, ^25^ ^]^ The conditions of differentiation can be found in **Table** **1**. All cell groups were positive for the β‐3 tubulin expression, which is a neuron‐specific marker protein (**Figure** **3**A).^[^ ^26^ ^]^ However, morphological analysis revealed differences among the groups (Figure 3B). The PVX‐RGD, PVX‐IKVAV, TMV‐RGD, and TMV‐IKVAV VNPs were designed to provide neuronal cells with adhesion sites and, therefore, are believed to be capable of replacing laminin. The number of neurites and branches, as well as neurite length, were similar for cells cultivated on laminin‐coated dishes and dishes coated with these VNPs. However, on PVX‐RGD‐coated dishes, the number of neurites (1.203 ± 0.736 per cell) and branches (0.625 ± 0.409 per cell) as well as the neurite length (3.278 ± 1.891 µm per cell) were slightly higher than those of cells on laminin‐coated dishes (number of neurites: 1.115 ± 0.542 per cell; number of branches: 0.557 ± 0.372 per cell; neurite length: 2.885 ± 0.991 µm per cell). Among VNP groups with laminin‐derived peptides, cells growing on TMV‐IKVAV‐coated dishes had the lowest number of neurites (0.724 ± 0.152 per cell) and branches (0.293 ± 0.096 per cell), and the shortest neurites (2.101 ± 0.128 µm per cell). Analysis of cell morphology showed that cells cultivated with PVX‐BDNF‐2A had fewer neurites (0.341 ± 0.160 per cell) and a similar number of branches (0.309 ± 0.167 per cell) compared to cells cultivated with conventional BDNF. However, the cells cultivated with PVX‐BDNF‐2A had significantly longer neurites (15.328 ± 6.723 µm per cell) than the cells cultivated with conventional BDNF (2.885 ± 0.991 µm per cell).

**Table 1:** Group Label Conditions.

Label	Condition
+LN	Laminin‐coated surface
‐LN	No laminin coating
+BDNF	BDNF was added to the medium
PVX‐RGD	Surface coated with PVX‐RGD instead of laminin
PVX‐IKVAV	Surface coated with PVX‐IKVAV instead of laminin
TMV‐RGD	Surface coated with TMV‐RGD instead of laminin
TMV‐IKVAV	Surface coated with TMV‐IKVAV instead of laminin
PVX wt	PVX wt added as a control, instead of BDNF
PVX‐BDNF‐2A	PVX‐BDNF‐2A added instead of BDNF

Figure 3C shows the changes in the gene expression levels of the neuronal markers RBFOX3, NCAM, and NSE after 22 days of differentiation with different VNPs. The neurospecific splicing gene RBFOX3^[^ ^27^, ^28^ ^]^ was expressed at significantly higher levels in cells cultivated with conventional BDNF on laminin‐coated dishes (+LN+BDNF) than in cells cultivated with PVX‐RGD, PVX‐IKVAV, TMV‐RGD, or TMV‐IKVAV instead of laminin. Cells cultivated without laminin exhibited levels of RBFOX3 expression similar to cells cultivated on laminin‐coated dishes. However, cells cultivated with PVX‐BDNF‐2A instead of BDNF exhibited significantly higher RBFOX3 expression than all the other groups. Cells cultivated without BDNF or PVX wt instead of BDNF expressed RBFOX3 at significantly lower levels than the cells cultivated in the presence of BDNF.

NCAM facilitates cell adhesion and neurite outgrowth and is downregulated during differentiation into mature neurons.^[^ ^29^, ^30^ ^]^ The NCAM expression level in cells cultured with PVX‐IKVAV and TMV‐IKVAV was significantly lower than in cells differentiated without laminin, but similar to cells differentiated on laminin‐coated dishes. NCAM expression levels were significantly higher in cells differentiated without BDNF and with PVX wt or PVX‐BDNF‐2A than in cells differentiated with conventional BDNF.

Neuron‐specific enolase is a marker for mature neurons.^[^ ^31^ ^]^ Its expression was significantly lower in cells differentiated on laminin‐coated dishes with conventional BDNF than in cells differentiated with PVX‐RGD, PVX‐IKVAV, or TMV‐RGD instead of laminin or PVX wt or PVX‐BDNF‐2A instead of BDNF.

Immunocytochemistry could not reveal differences in maturity between neuronal cells differentiated under the different conditions. Cells differentiated with PVX‐IKVAV, PVX‐RGD, TMV‐IKVAV, and TMV‐RGD were stained with antibodies against the neuronal markers β‐3 tubulin, MAP2, and NSE. However, there was no difference in expression intensities visible (Figures S3–S5, Supporting Information).

### 2.4 Alignment of Plant Virus Nanoparticles and Guidance of Neuronal Outgrowth

**Figure 4:**
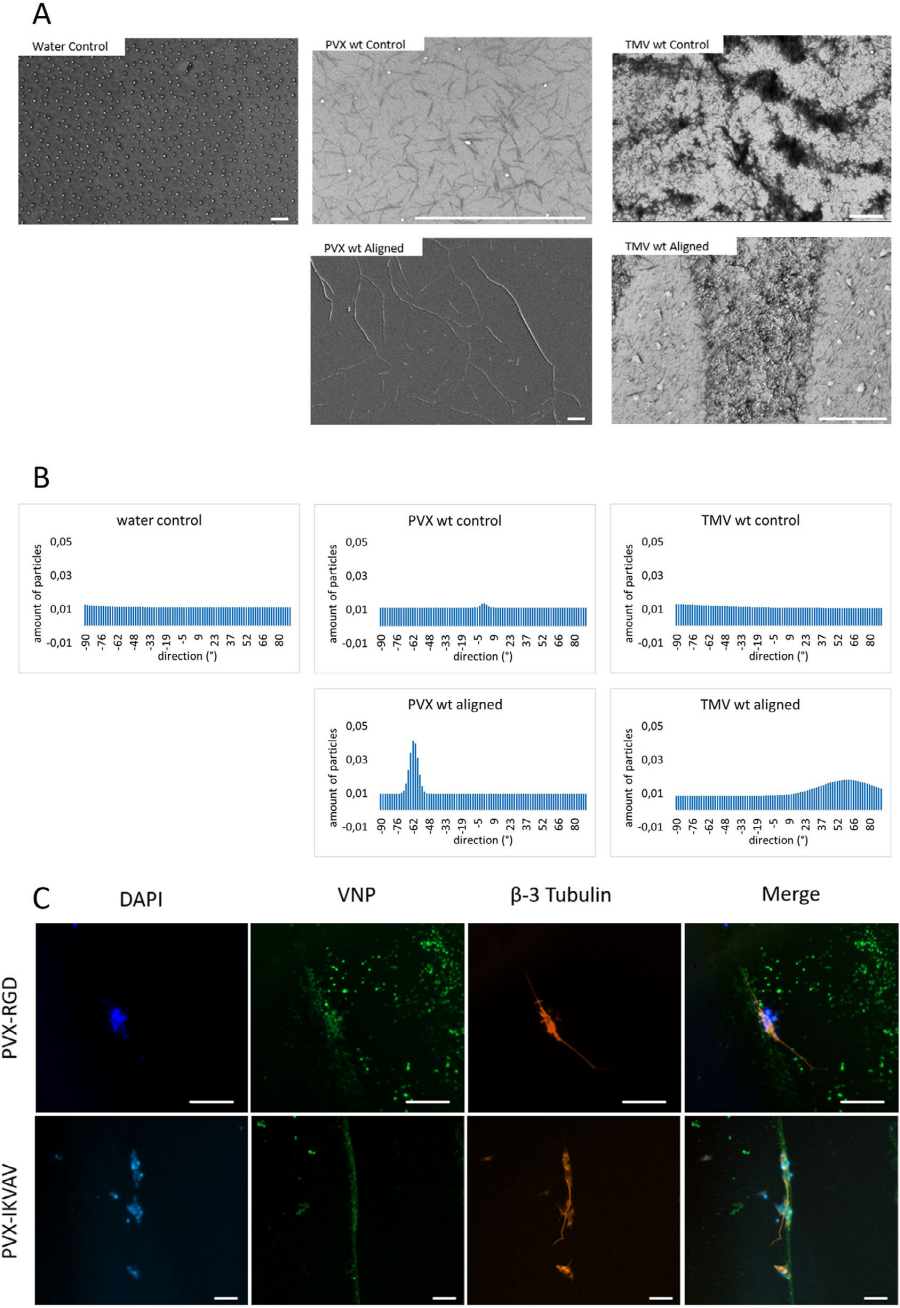
Alignment of VNPs and growth guidance of neurons. A) SEM images of orientated PVX and TMV particles. Water served as a negative control. VNP controls were not oriented but pipetted onto the surface without printed orientation channels. Scale bars represent 5 µm. B) Directionality histograms of the SEM images of aligned and non‐aligned VNP particles. The *X‐*axis represents the orientation angle, and the *Y*‐axis represents the number of particles oriented in the specific direction. C) Immunostaining of neurons on day 22 with DAPI (blue), the neuronal marker β‐3 tubulin (red), and anti‐PVX CP antibody (green). Cells were differentiated from day 10 onward, on orientated PVX‐RGD and PVX‐IKVAV nanoparticles. Scale bar represents 50 µm.

Scanning electron microscopy was used to visualize the VNPs and investigate their degree of orientation after alignment (**Figure** **4**A). In the control group, water was used for the alignment approach with very narrow channels. Since no rod‐like particles were present in the SEM image of the control group, the possibility of pluronic residues in the image could be excluded. Thus, it was certain that the aligned structures visible in the electron microscopy images came from the VNPs and not from the alignment approach itself. Electron microscopy images of the PVX and TMV control groups showed no aligned structures. SEM images also showed that aligned PVX nanoparticles adhered to each other, creating larger structures than the individual nanoparticles. Conversely, the PVX nanoparticle controls resembled a network, but the individual particles could be distinguished. Further quantification of nanoparticle orientation using the directionality histogram tool in *Fiji* revealed that the particles in the aligned PVX and aligned TMV groups were not randomly distributed but rather orientated in a specific direction (Figure 4B). Additionally, the directionality histograms revealed that PVX nanoparticle orientation was more prevalent than TMV nanoparticle orientation.

Because interaction with attachment peptides was more prevalent in the PVX groups, neurodifferentiation was performed on orientated PVX‐RGD and PVX‐IKVAV nanoparticles starting on day 10 of differentiation. Figure 4C shows the differentiated neurons on day 22. The direction of neuron growth followed that of the aligned PVX‐RGD and PVX‐IKVAV nanoparticles, respectively. Neurodifferentiation was confirmed by the β‐3 tubulin marker in both groups. Aligned PVX wt VNPs were also used in this experiment. However, due to the lack of adhesion molecules, the cells did not attach to the dish surface.

## 3 Discussion

Functionalized plant VNPs have emerged as versatile platforms for various biomedical applications due to their inherent biocompatibility, ease of modification, and ability to display bioactive molecules. These properties make VNPs promising candidates for drug delivery, vaccine development, and tissue engineering.^[^ ^32^ ^]^ This study investigated several plant VNPs for their ability to present the attachment or signaling peptides to promote the neurodifferentiation of SH‐SY5Y cells. Plant VNPs that present RGD motifs on their surfaces have been extensively researched with human mesenchymal stem cells and have been proven to promote the process of osteogenesis.^[^ ^5^, ^15^, ^18^, ^33^ ^]^ However, most of these studies focused only on the final cellular responses without investigating the mechanism of cell–VNP interaction in detail, even though Lin et al.^[^ ^33^ ^]^ demonstrated some interaction through immunostaining. To the best of our knowledge, this is the first study to prove the interaction between the VNP and the corresponding cellular receptor. Findings from Schuphan et al.^[^ ^18^ ^]^ suggested that the PVX VNPs provide a more suitable platform for peptide presentation than TMV and that the higher availability for cell interaction with the peptide is due to the virus's flexibility rather than a higher total number of peptides as presented on TMV. Both PVX‐RGD and TMV‐RGD were shown to be cytocompatible and to enhance the adhesion of mesenchymal stromal cells and mouse neuronal crest cells, respectively.^[^ ^15^, ^16^ ^]^ In the present study, we demonstrated the presence of VNPs on the surfaces of the dishes using immunostaining. Interactions of PVX‐RGD, PVX‐IKVAV, and TMV‐RGD, and the differentiating SH‐SY5Y cells were visible in immunostained images (Figure 2). To better understand the interaction mechanism, immunoprecipitation with the most probable interacting receptors^[^ ^34^ ^]^ was performed. While immunoprecipitation confirmed the interaction between PVX‐IKVAV and the integrin‐β1 receptor, this was not the case for PVX‐RGD, TMV‐RGD, or TMV‐IKVAV. However, RGD binds not only to the β1‐integrin subunit, but also to other integrin receptors.^[^ ^34^, ^35^ ^]^ Therefore, the interaction of PVX‐RGD with the cells could be via another integrin receptor, which should be tested by immunoprecipitation in future studies. Immunoprecipitation could not confirm the interaction of TMV‐RGD or TMV‐IKVAV with the integrin‐β1 receptor, even though TMV‐RGD interaction with the cells was visible in immunostained images. This could be due to RGD binding to integrin‐subunits other than the integrin‐β1 subunit, which aligns with immunostaining results showing no interaction between TMV‐IKVAV and the cells. Nevertheless, since IKVAV only binds to the integrin‐β1 subunit an interaction between TMV‐IKVAV and the SH‐SY5Y cells can be ruled out, which is consistent with the immunostaining results. Due to TMV's more rigid structure and a tighter packing of the CP subunits that present the peptides, the functional peptide is probably unavailable to cellular receptors. This result is supported by the findings of Schuphan et al.,^[^ ^18^ ^]^ as the same peptide had a greater influence on cells when presented on the flexible PVX.

In addition to investigations of VNP–cell interactions, we examined subsequent intracellular pathway activation upon the recognition of the presented peptides. When binding to the integrin receptor, the intracellular FAK pathway is activated, and FAK becomes phosphorylated.^[^ ^35^, ^36^ ^]^ Phosphorylated FAK (pFAK) was present in all groups, even when no functional peptides were exposed to the cells. This may be because FAK is phosphorylated not only upon binding to laminin‐derived peptides but also following mechanical stress and growth factors.^[^ ^36^, ^37^ ^]^ While the bands at 140 kDa were present in both No VNP and PVX wt group, in the lanes with samples from PVX‐RGD and PVX‐IKVAV treated cells, they were weaker or completely absent (Figure 2E–G). However, lower molecular weight FAK isomers were getting phosphorylated, at 100 and 40 kDa for PVX‐RGD and PVX‐IKVAV, respectively. To the best of the author's knowledge, no previous examination of FAK activation by VNPs has been conducted by other researchers. Therefore, further studies focusing on the mechanism of the downstream signaling from different VNPs are necessary. As FAK is involved in multiple pathways,^[^ ^34^, ^36^, ^37^ ^]^ different protein activations, for example, phosphorylation of Src‐family protein tyrosine kinases (SFKs) and integrin‐linked kinase (ILK),^[^ ^34^ ^]^ should be investigated to better understand the mechanism of action of VNPs on SH‐SY5Y differentiation.

The differentiated SH‐SY5Y cells were not fully mature after 22 days as the expression of the late neuronal differentiation marker NSE was rather low in all groups. This is in agreement with the results of Cheung et al., who did not find an upregulation of the late neuronal differentiation marker protein MAP2 during the differentiation of SH‐SY5Y cells.^[^ ^38^ ^]^ Even though the expression of NSE was low in all groups, it was upregulated from day one to day 22 of differentiation, which is in agreement with the findings of other studies.^[^ ^38^, ^39^ ^]^ Cells that were differentiated with VNPs had even higher NSE expression levels than cells differentiated with BDNF and laminin, which warrants further investigation. However, according to RBFOX3 expression, a marker for late neuronal development and maturation,^[^ ^27^, ^28^, ^40^, ^41^ ^]^ the differentiated cells from the laminin and PVX‐BDNF‐2A groups were mature neurons. The gene level of RBFOX3 in the PVX‐IKVAV group was also relatively high, which corresponds to the results from immunoprecipitation. Nevertheless, the suitability of PVX‐IKVAV as a replacement for laminin in neuronal differentiation should be tested in further studies with higher concentrations of PVX‐IKVAV, as well as its combinations with PVX‐RGD or other VNPs displaying laminin‐derived peptides, such as YIGSR or RNIAEIIKDI. Additionally, the early neuronal marker NCAM was downregulated in cells differentiated with laminin compared to cells growing on uncoated dishes. The expressions of NCAM in cells that were differentiated in the presence of PVX‐RGD, PVX‐IKVAV, TMV‐IKVAV, and PVX‐BDNF‐2A were similar to the expression levels of NCAM in cells growing on laminin. It can be therefore concluded that these cells were in a similar state of maturity. PVX‐IKVAV and PVX‐BDNF‐2A were found to interact with the cells via immunoprecipitation, but the interaction of PVX‐RGD, TMV‐RGD, and TMV‐IKVAV could not be shown. It can be inferred that PVX‐RGD probably interacted with other integrin‐subtype receptors, as has been shown by Lauria et al.^[^ ^15^ ^]^ Why the expression of NCAM was lower in cells of the TMV‐IKVAV group remains unclear. But since the expression of NCAM was first up‐ and then downregulated, it may be that these cells were in a very early state of differentiation. The β‐3 tubulin protein expression was upregulated from day 10 to day 22 and had the same levels for all the groups. As it is an early neurogenesis marker, it seems to be independent from laminin or BDNF.

The morphology of the neurons differentiated on laminin‐coated dishes was similar to those differentiated on PVX‐RGD, PVX‐IKVAV, TMV‐RGD, or TMV‐IKVAV‐coated dishes or uncoated dishes, which concludes that laminin does not have a significant effect on neurite number, length, or the number of branches. However, with the presence of PVX‐BDNF‐2A neurons had significantly longer neurites, while the number of neurites was smaller compared to neuronal cells differentiated with conventional BDNF. This goes along with the findings reviewed by Deinhardt and Chao^[^ ^42^ ^]^ who state that by signaling through the TrkB receptor BDNF promotes neurite outgrowth and branching. However, at the sudden withdrawal of BDNF respective neurites get pruned. By using PVX‐BDNF‐2A, a low overall but very high local concentration of BDNF is present in the suspension. This leads to the assumption that neurites which are in contact with PVX‐BDNF‐2A grow along these nanoparticles and extend further while other neurites which grow in different directions and lack direct contact to PVX‐BDNF‐2A nanoparticles stop growing and die back. This assumption should be further investigated in additional studies. In summary, this protocol can be used to differentiate SH‐SY5Y cells into relatively mature neurons. PVX‐BDNF‐2A can replace BDNF, and PVX‐IKVAV and PVX‐RGD alone can partially replace laminin. However, further studies should test PVX‐IKVAV and PVX‐RGD at higher concentrations, as well as their combined use. The differentiated neuroblastoma cells exhibited typical neuronal morphology regardless of laminin presence. This aligns with the findings of Feng et al. who found no significant morphological differences between mouse neuronal crest cells cultivated on functional TMV CPs or TMV wt.^[^ ^16^ ^]^ In contrast, Lauria et al. showed that PVX‐RGD enhances cell attachment and spreading in mesenchymal stromal cells.^[^ ^15^ ^]^ The neuritic length and branches depended upon the presence of BDNF. PVX‐BDNF‐2A produced much longer neurites than conventional BDNF. Nevertheless, cells cultured with PVX‐RGD and PVX‐IKVAV also exhibited neuronal phenotypes, even when they were a little less mature. The cells seem to need more functional sites from laminin than just the short sequences RGD or IKVAV. In addition to these, laminin contains the very important sequence YIGSR, which has already been implemented into hydrogels.^[^ ^9^ ^]^ Therefore, the combination of PVX‐RGD, PVX‐IKVAV, and PVX‐YIGSR could be a promising strategy to differentiate SH‐SY5Y cells toward neurons. Schense et al. demonstrated that four laminin‐derived peptides (RGD, IKVAV, YIGSR, and RNIAEIIKDI) and their different combinations can have an inhibitory, additive, or synergistic effect on the neurite extensions.^[^ ^43^ ^]^ Therefore, to fully substitute laminin, a combination of these four peptides needs to be present during neurodifferentiation of cells. However, genetic fusion of the YIGSR sequence to the PVX CP, either directly via a flexible glycine‐serine‐rich (G_4_S)_3_ linker or with the FMDV 2A ribosomal skipping sequence, did not yield in the production of VNPs as evidenced by the absence of systemic symptoms in plants at 20 dpi (Figure S8, Supporting Information). This indicates that the introduced CP modification interfered with PVX assembly, stability, or cell‐to‐cell movement. To nonetheless enable presentation of the YIGSR motif on PVX particles, a plug‐and‐display approach such as SpyTag/SpyCatcher can be employed.^[^ ^44^ ^]^

Moreover, we showed that PVX‐BDNF‐2A could fully replace BDNF, and cells had the same or even higher state of maturity. This has never been tested before and confirms that the CPs of the PVX nanoparticles are accessible to the cells. Additionally, a cost reduction of approximately eightfold can be achieved by using PVX‐BDNF‐2A, based on the calculated costs of 20 € µg^−1^ BDNF versus the estimated cost of ≈2.5 € µg^−1^ for the production of PVX. The calculations presented herein are based on the animal‐free production of wild‐type PVX in plants, which usually demonstrates a comparatively higher yield than that of modified VNPs such as PVX‐BDNF‐2A. It is important to note that due to the locally increased concentration of the functional peptides displayed on PVX, a reduced amount of VNPs is required to elicit a positive biological effect. Furthermore, the filamentous structure of PVX confers the advantage of enabling specific orientation of VNPs through shear forces.

Neuronal cells thrive and develop in micro‐organized environments that mimic their natural surroundings within the nervous system. The growth and orientation of neurites can be guided by physical and biological cues.^[^ ^3^, ^4^ ^]^ Furthermore, the directed outgrowth of neurites is essential for neuronal tissue engineering and peripheral nerve replacement.^[^ ^16^, ^45^, ^46^, ^47^ ^]^ The PVX VNPs build this microarchitecture by forming network‐like structures.^[^ ^15^ ^]^ The present study showed that PVX nanoparticles can be aligned by shear stresses. Previous studies have demonstrated this alignment with TMV and other nanoparticles.^[^ ^5^, ^16^ ^]^ However, this study provides the first proof that the alignment is also possible with PVX using a novel, in‐house developed biofabrication method with sacrificial materials. The neuritic outgrowth of the cells followed the aligned PVX‐RGD and PVX‐IKVAV nanoparticles, as demonstrated by immunostaining. This finding aligns with previous research on neuronal guidance using viral nanoparticles. Notably, Feng et al. successfully demonstrated neuronal orientation using aligned TMV nanoparticles within capillary tubes.^[^ ^16^ ^]^ In their study, neuronal alignment was observed within these capillaries, even with wild‐type TMV particles. However, it is important to note that their experimental design differs significantly from the current study because they used a capillary‐based system rather than aligned strands of VNPs.

## 4 Conclusion

This study demonstrates that plant VNPs, particularly PVX presenting functional peptides, effectively enhance the neurodifferentiation of SH‐SY5Y cells. We present the first direct evidence of specific interactions between PVX‐IKVAV and the integrin β‐1 receptor and between PVX‐BDNF‐2A and the trk receptor. We also confirm the activation of subsequent intracellular pathways. Notably, PVX‐BDNF‐2A outperformed conventional BDNF in promoting neurite outgrowth and neuronal maturation, highlighting its potential in neuronal tissue engineering. While PVX‐RGD likely interacts with other integrin subunits, TMV‐based nanoparticles showed limited peptide accessibility, which is assumed to be due to their rigid structure. Furthermore, aligned PVX‐RGD and PVX‐IKVAV nanoparticles successfully guided neurite outgrowth. Overall, PVX nanoparticles functionalized with peptides and growth factors are a promising modular tool for neuroregenerative applications.

## 5 Experimental Section

### Design of Viral Vectors

The IKVAV encoding sequence was fused to the 5′ end of the PVX *CP* gene via PCR with GoTaq G2 DNA Polymerase (Promega, Mannheim, Germany) and specific primers IKVAV‐CP and M13‐uni (Table S1, Supporting Information). This introduced an EagI or SalI site at the 5′ or 3′ end. The vector pTCXIIc served as a template.^[^ ^48^ ^]^ GoTaq G2 DNA Polymerase (Promega) was used to 3′ adenylate the PCR product, prior to transferring it to the plasmid pCR2.1 TOPO (Thermo Fisher Scientific, Dreieich, Germany) and subsequently transforming chemically competent TOP10 cells, in accordance with the manufacturer's guidelines. The integrity of the product was confirmed by PCR using primers M13‐fw and M13‐uni. After digestion with EagI and SalI (New England Biolabs, Frankfurt am Main, Germany), the IKVAV encoding sequence was inserted into the pTCXIIc vector^[^ ^48^ ^]^ which was linearized with the same restriction enzymes and dephosphorylated with calf intestinal alkaline phosphatase (New England Biolabs) to prevent auto‐ligation. Ligation was carried out at 16 °C overnight using T4 DNA ligase (Promega), followed by precipitation with glycogen/ethanol and electroporation of *E. coli* DH5α cells. The transformation was confirmed by colony PCR using primers CX2 and CX1.

Cloning of the pTMV‐IKVAV was achieved by fusing the IKVAV encoding sequence to the 3′ end of the TMV *CP* gene, using Pfu DNA Polymerase (Promega) and specific primers GA_30B_fwd and 30B‐IKVAV. The vector pTMV‐RGD^[^ ^18^ ^]^ served as a template. This vector was linearized using the PacI and NotI restriction enzymes, and NEBuilder HiFi DNA Assembly Master Mix (New England Biolabs) was employed to assemble the plasmid and PCR product according to the manufacturer's recommendations. Colony PCR using primers TMV5482f and TMV6269r confirmed the transformation.

The BDNF encoding sequence was fused to the 5′ end of the PVX *CP* gene by PCR using Pfu Polymerase (Promega) and specific primers BDNF‐2A_fw and GA_CP‐PVX_rev. The vector pPVX‐HABP‐CP^[^ ^33^ ^]^ served as a template. The pPVX‐BDNF‐2A vector was cloned using Gibson assembly as described above. It was noteworthy that BDNF was fused using a foot‐and‐mouth disease virus (FMDV) 2A sequence, which was demonstrated to cause ribosomal skipping during expression, resulting in only 25% of the envelope proteins carrying the functional peptide.^[^ ^23^ ^]^ The primers CX3 and CX8 were used in colony PCR to confirm the transformation.

The resulting vectors pPVX‐IKVAV, pTMV‐IKVAV, and pPVX‐BDNF‐2A were verified by sequencing (Eurofins Genomics, Ebersberg, Germany). The viral vectors pPVX‐RGD^[^ ^15^ ^]^ and pTMV‐RGD^[^ ^18^ ^]^ were designed in previous studies. Sequences are displayed in Section S1 (Supporting Information).

### Infection and Cultivation of *N. benthamiana*

The infection and cultivation of *N. benthamiana* plants was carried out as described in Schuphan et al.^[^ ^18^ ^]^

### Purification of Biomimetic VNPs

The VNPs were extracted from infected leaf material and subsequently purified via ultracentrifugation, as previously described.^[^ ^33^ ^]^ The purified VNPs were stored in 1.5‐mL low‐protein‐binding tubes (Thermo Fisher Scientific).

### Characterization of Biomimetic VNPs

Infected leaf material was homogenized in 2× vol of 1× PBS following centrifugation (20,000× g, 10 min, 4 °C). Then, the supernatants (15 µL) or purified VNPs (5 µg) were boiled for 5 min in 5× reducing loading buffer before being separated by SDS‐PAGE on a 12% resolving gel and a 4% stacking gel 27. P7719 Pre‐stained Protein Standards (New England Biolabs) were used for sizing. The separated proteins were either stained with Coomassie Brilliant Blue or transferred to a Hybond‐C nitrocellulose membrane (GE Healthcare Life Sciences) using a semi‐dry blotting system (BioRad Laboratories, Munich, Germany) for western blot analysis. The membrane was blocked for at least 45 min in 4% (w/v) skimmed milk in PBS and incubated overnight at room temperature with a primary polyclonal anti‐PVX CP (DSMZ, Braunschweig, Germany) or anti‐TMGMV CP antibody, described here as an anti‐TMV CP antibody (DSMZ) diluted 1:5,000 in PBS. This was followed by incubation for at least 3 h with an alkaline phosphatase (AP)‐labeled goat‐anti rabbit (GAR^AP^) secondary antibody (Dianova, Hamburg, Germany) diluted 1:5,000 in PBS. The signal was visualized by staining with nitroblue tetrazolium chloride/5‐bromo‐4‐chloro‐3‐indolyphosphate p‐toluidine salt (Carl Roth, Karlsruhe, Germany).

The target sequence fused to the TMV or PVX *CP* gene was confirmed by analyzing the viral RNA. To that end, 2 µm of either Oligo‐dT or the TMV_RT_rev primer was added to 5 µg of purified VNPs. The mixture was heated to 80 °C for 10 min and then chilled on ice for 10 min, to allow primer annealing. Reverse transcription was carried out using M‐MLV Reverse Transcriptase RNase H Minus Point Mutant (Promega). For cDNA synthesis, the following were added: 5 µL of 5× M‐MLV reaction buffer, 1 mm of dNTPs, 2.5 µL of DEPC‐treated water, and 1 µL of M‐MLV reverse transcriptase. The mixture was then incubated for 30 min at 40 °C, followed by 20‐min incubations at temperatures of 45, 50, 55, and 70 °C, respectively. The integrity of the *CP* fusion was determined by PCR using PVX‐ (CX3 and CX8) or TMV‐specific primers (TMV_seq_fw and TMV_RT_rev).

To visualize the VNPs, pioloform‐coated nickel‐grids (Plano, Wetzlar, Germany) were floated on VNPs (final concentration of 0.4 mg mL^−1^) for 2—3 h at room temperature. After washing with water, the grids were contrasted with 1% (v/v) phosphotungstic acid and examined using a Zeiss LEO 906E TEM (Zeiss) operating at an accelerating voltage of 60 kV.

### Cell Culture and Differentiation

The SH‐SY5Y neuroblastoma cell line was obtained from the DSMZ. The cells were maintained in RPMI medium (RPMI 1640, Life Technologies, Carlsbad, USA) supplemented with 1% (v/v) penicillin/streptomycin (Pan‐Biotech, Aidenbach, Germany) and 10% (v/v) fetal calf serum (FCS, Gibco by Life Technologies, Carlsbad, USA) in a 5% (v/v) CO_2_ atmosphere at 37 °C. SH‐SY5Y cells were differentiated into neuronal cells within 22 days according to the protocol proposed by Shipley et al.^[^ ^49^ ^]^ Briefly, the cells were cultured in Eagle's Minimum Essential Medium (30‐2003, ATCC, Manassas, USA) supplemented with 2.3% (v/v) heat‐inactivated FCS, 1% (v/v) penicillin‐streptomycin, 1% (v/v) *L*‐Glutamine (Sigma G7513, Sigma Aldrich, St Louis, USA), and 10 µm retinoic acid for seven days. Then, the concentration of heat‐inactivated FCS in the medium was reduced to 1% (v/v), and the cells were cultured in this medium for three days. On day 10 of differentiation, the cells were transferred to surfaces coated with either laminin (0.5 µg cm^−^^2^, rh‐Laminin 521, Life Technologies) or virus (40 µg cm^−^^2^, PVX‐RGD, PVX‐IKVAV, PVX wt, TMV‐RGD, TMV‐IKVAV, TMV wt) surfaces. Starting on day 11, the cells were cultured in Neurobasal Medium supplemented with KCl (40 µm), B27 supplement (2% v/v), retinoic acid (10 µm), BDNF (50 ng mL^−1^, BDNF 450‐02, Peprotech, Hamburg) or PVX‐BDNF‐2A (50 µg mL^−1^), and db‐cAMP (980 µg mL^−1^, StemCell Technologies, Vancouver, Canada). At day 22, the cells were differentiated and further analyzed by immunofluorescence imaging, qPCR, or western blot.

### Quantitative PCR of Neuronal Differentiation Genes

Samples for gene expression analysis were obtained from the differentiated cells after 22 days. RNA was extracted from the cells using the RNeasy Mini‐Kit (Qiagen, Hilden, Germany), according to the manufacturer's protocol. The extracted RNA was then used to synthesize cDNA via reverse transcription using the first‐strand cDNA synthesis kit (K1612, Thermo Fisher Scientific) according to the manufacturer's protocol. The concentration of the synthesized cDNA was measured with the QuantiFluor dsDNA System (Promega, USA), set to 1 ng µL^−1^, and further used for qPCR. Primers for the genes of interest were designed using Primer‐BLAST software^[^ ^50^ ^]^ and obtained from Eurofins Genomics (Eurofins Genomics, Luxembourg). The QuantiNova SYBR Green PCR Kit (Qiagen) was used for the qPCR assay with the reagent volumes defined in Table S2 (Supporting Information). Samples were tested in triplicate for the neuronal markers NCAM1, NSE, and RBFOX3. Glyceraldehyde 3‐phosphate dehydrogenase (GAPDH) was used as the housekeeping gene. The corresponding primer sequences are specified in Table S1 (Supporting Information). The ΔΔCt method was used to calculate the fold changes in gene expression. Cells cultivated on laminin‐coated and uncoated dishes were used as positive and negative controls, respectively.

### Immunofluorescence Microscopy

After 22 days of neurodifferentiation, the samples were fixed with 4% paraformaldehyde (PFA, Thermo Fisher Scientific) for 30 min at room temperature, after which they were washed with PBS. Next, a blocking and permeabilization solution containing 1% (w/v) bovine serum albumin (BSA) and 0.1% (v/v) Triton X‐100 (Sigma Aldrich) in PBS was added to the samples for 30 min. A solution containing 0.2% BSA in PBS was used for antibody incubation and washing steps. Primary antibodies were diluted according to Table S3 (Supporting Information). After an overnight incubation at 4 °C with the primary antibodies, the samples were washed three times for five minutes. Then, secondary antibodies were added to the samples, which were then incubated for two hours at room temperature. The samples were additionally stained for one minute with a 1:2000 dilution of 4′,6‐diamidin‐2‐phenylindol (DAPI, Thermo Fisher Scientific) in PBS. The stained samples were imaged with a fluorescence microscope (Axio imager M2, Zeiss, Oberkochen, Germany).

### Cell Morphology Analysis

Image analysis was performed using Fiji software^[^ ^51^ ^]^ and its SNT plugin.^[^ ^52^ ^]^ The neurites labeled with anti‐β‐3 tubulin in each image were traced and analyzed for length, number of branches, and number of neurites, and compared based on the number of cells visible in the image (Figure S7, Supporting Information).

### Pathway Activation Studies

Undifferentiated SH‐SY5Y cells were used to investigate downstream pathway activation from integrin β‐1. The cells were cultivated in T175 flasks until they reached an average confluence of 90%. Then, they were treated with VNPs at a concentration of 50 µg mL^−1^ for 30 min. After incubation, the cells were washed with PBS, detached from the surface with a 0.05% (v/v) trypsin solution (Gibco Trypsin‐EDTA (0.05%), Thermo Fisher Scientific, Schwerte), pelleted, and placed on ice. The pellets were lysed with a lysis buffer (Cell Extraction Buffer, Thermo Fisher Scientific), supplemented with 1× proteinase and phosphatase inhibitors (Halt Protease and Phosphatase Inhibitor Cocktail (100×), Thermo Fisher Scientific). After a 30‐min incubation on ice, the lysates were centrifuged at 13,000 g and 4 °C for 10 min, and the supernatants were collected. Then, the samples were prepared for SDS‐PAGE and western blotting.

### Gel Electrophoresis and Western Blot of Neuronal Markers

Protein concentrations in cell lysates were measured using the Pierce BCA Protein Assay Kit (Thermo Fischer Scientific) and a microplate reader (SpectraMax M2 Fluor, Molecular Devices, San Jose, USA), according to the manufacturer's protocol. The cell lysate volume was calculated to contain 15 µg of total protein, which was then mixed at a ratio of 3:1 with loading buffer (450 µL of 4× Laemmli sample buffer (BioRad, Hercules, USA) and 50 µL of 2‐β‐mercaptoethanol (Sigma Aldrich). The samples and a molecular weight ladder (Spectra Multicolor Broad Range Protein Ladder, 10–260 kDa, Thermo Fisher Scientific) were pipetted into a precast gel (Criterion TGX Precast 4–20%, BioRad), placed in a gel electrophoresis chamber (BioRad, Hercules, USA), and filled with running buffer (100 mL 10× Tris/Glycine/SDS Buffer (BioRad) + 900 mL distilled water). To transfer the proteins from the gel to a PVDF membrane, the Trans‐Blot Turbo System and the corresponding RTA Transfer Kit (BioRad) were used. After blotting, the membrane was blocked with 5% (w/v) skimmed milk (Carl Roth) in PBST buffer (PBS with 0.1% (v/v) Tween‐20) for 1 h. Then, it was incubated with a primary antibody solution containing anti‐FAK, anti‐pFAK, anti‐MAPK (Erk1/2) (Santa Cruz Biotechnology, Dallas, USA), anti‐PVX, anti‐TMV (DSMZ), anti‐trk, anti‐pMAPK (Erk1/2) (Cell Signaling Technology, Danvers, USA), or anti‐Actin (MP Biomedicals, Irvine, USA) over‐night at 4 °C. After washing, the membrane was incubated with the HRP‐conjugated secondary antibody for 1 h at room temperature. For visualization, the membranes were prepared using the ECL Advance Western Blotting Detection Kit (GE Healthcare, Chicago, USA) and imaged using the iBright CL 1500 Imaging System (Thermo Fisher Scientific). The images obtained were analyzed using Fiji.^[^ ^51^ ^]^

### Immunoprecipitation

To confirm the mechanism of virus–cell interactions, undifferentiated SH‐SY5Y cells were incubated with VNPs (PVX wt, PVX‐RGD, PVX‐IKVAV, PVX‐BDNF, TMV wt, TMV‐RGD, or TMV‐IKVAV) at a concentration of 50 µg mL^−1^ for three hours. Cells with no contact to VNPs were used as a negative control. Cytosolic and membrane protein fractions were extracted using the Mem‐Per Plus Kit (Thermo Fisher Scientific) according to the manufacturer's protocol. The isolated membrane proteins were then incubated with primary antibodies in the 1.5‐mL low‐protein‐binding tubes (Thermo Fisher Scientific) at 4 °C on a rotating platform. The proteins from the cells that were incubated with VNPs carrying laminin‐derived peptides were incubated with mouse anti‐integrin β‐1 (Santa Cruz Biotechnology), and the proteins from the cells incubated with PVX‐BDNF‐2A were labeled with rabbit anti‐Trk (Cell Signaling Technology). To serve as a technical control, the proteins were incubated with IgG to identify bands on the western blot membrane that were caused by nonspecific interactions or the IgG itself. The antibody–receptor complex was then placed with µMACS Protein G MicroBeads (Miltenyi Biotec, Bergisch Gladbach, Germany) for 1 h on ice. Then, the solution containing the MicroBead–antibody–receptor complex was loaded onto µColumns (Miltenyi Biotec), which was positioned on a µMACS Separator on a MACS MultiStand beforehand. The complex was isolated according to the manufacturer's protocol (Section S3, Supporting Information). The isolated samples were collected and used for further gel electrophoresis and western blotting. The presence of PVX CP was investigated in the 25‐kDa bands, and the presence of TMV CP was investigated in the 17.5‐kDa.

### Nanoparticle Alignment

**Figure 5:**
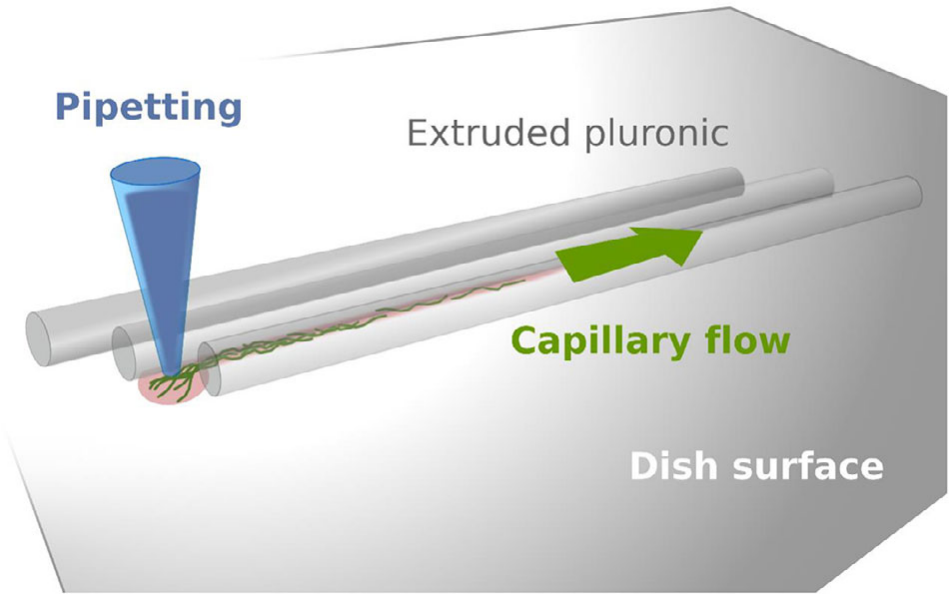
Principle of creating channels for alignment of VNPs. The illustration shows the method of aligning VNPs. The pluronic strands were extruded using a 3D printer. The viral nanoparticles were pipetted to the inlets and were sucked into the channels between the pluronic and the dish surface by capillary forces.

Sterile 35% (w/v) pluronic (pluronic F‐127 powder, Sigma Aldrich) was 3D‐printed using the extrusion method with a BioX Bioprinter (Celllink, Gothenborg, Sweden). Pluronic F‐127 was extensively used in the literature for precise 3D bioprinting in tissue engineering approaches as well as the sacrificial bioink to create complex tissue‐like structures.^[^ ^53^, ^54^, ^55^ ^]^ A conical needle with an inner diameter of 0.2 mm (27 G) (Nordson EFD, Feldkirchen, Germany) was used to print three 10‐mm‐long strands, 0.5 mm apart. The pluronic lines were printed with an offset to create entrances into the capillaries, which were formed by two pluronic strands and the dish's surface. The VNP solution was pipetted close to the capillary entrances and sucked into the capillaries by capillary forces (**Figure** **5**, Video S1, Supporting Information). The aligned VNPs were left to dry overnight, after which the pluronic strands were washed away with ice‐cold PBS. A silicon surface was used for samples intended for scanning electron microscopy investigations, and a cell culture dish was used for cell alignment investigations. Cells were seeded on top of the aligned VNPs on day 10 of differentiation and cultured for an additional 11 days, adhering to the differentiation protocol previously described. Finally, the samples were fixed and stained according to the immunostaining protocol (see above).

### Scanning Electron Microscopy

Samples were prepared on silicon wafers for scanning electron microscopy. Prior to imaging, the samples were fixed in 3% (v/v) glutaraldehyde (Agarscientific, Wetzlar, Germany) and washed in 0.1 m Sorensen's Phosphate Buffer (Merck) for 15 min. The samples were dehydrated by placing them in an ascending dilution of ethanol (30% (v/v), 50% (v/v), 70% (v/v), 90% (v/v), and 3× 100% (v/v)) for 10 min each and then drying them using a critical point drying method in liquid CO_2_ (CriticalPointDryer, Polaron, Quorum Technologies Ltd, Ashford, England). Scanning electron microscopy was performed in a high vacuum environment (Quattro S, Thermo Fisher Scientific) with a beam current of 0.17 nA and an acceleration voltage of 5 kV. The Quattro S was funded by the Deutsche Forschungsgemeinschaft (DFG, 495 328 185).

### Statistical Analysis

The results were presented as mean ± standard deviations. All analyses were conducted based on at least three independent samples. Exact sample sizes were indicated in the figure descriptions. Gene expression data from qPCR were normalized to GAPDH expression of the same group and the D0 positive control of the same gene. Data analysis of neuronal morphology and gene expression was performed using one‐way analysis of variance. Statistical significance was defined as *p *≤ 0.05 (*), *p *≤ 0.01 (**), *p *≤ 0.001 (**), *p *≤ 0.0001 (***), or *p *≤ 0.00001 (****). Statistical analysis was performed using the software Graphpad Prism version 10.0.0 for Windows (Graphpad Software Boston, Massachusetts USA, www.graphpad.com).

## Conflict of Interest

The authors declare no conflict of interest.

## Supporting information



Supporting Information

Supplemental Movie 1

## Data Availability

The data that support the findings of this study are available from the corresponding author upon reasonable request.
