# Supplementary material for: Plant‐Derived Viral Nanoparticles Enable Simultaneous Guidance of Neuronal Cell Outgrowth and Targeting of Neurodifferentiation Pathways
Source: Small. 2025 Nov 21;22(1):e09395. doi: 10.1002/smll.202509395 (PMC12757991; doi:10.1002/smll.202509395)
Supplement: Supplementary file 1 — Supporting Information [file SMLL-22-e09395-s002.docx]

**Plant-derived viral nanoparticles enable simultaneous guidance of neuronal cell outgrowth and targeting of neurodifferentiation pathways**

*Mira Ritter, Natalija Stojanović, Simon Zschieschang, Johannes Grader, MHD Naeem Assasa, Eva Miriam Buhl, Andrea Coschiera, Stefan Schillberg, Juliane Schuphan*, Horst Fischer**

Supporting Information

**Table S1.** Primer sequences.

| **Primer name** | **Sequence (5′-3′)** |
| --- | --- |
| 30B-IKVAV_rev | ACCTCAAGTTGCAGGACCGCGGCCGCTTATCTATCAGCAGAAACAGCAACCTTAATAGATGCAGCAGTAGCCGGAGTTGTGGTC |
| BDNF-2A_fwd | TACTCGAAAGAGGTCAGCACCAGCTAGCGCTACCGGTCGCCACCATGCGAGGCATCGACAAACGTCATTGGAACTCACAAGGAGGCTCCGGATCTAGAAATTTTG |
| CX1 | TTGAAGAAGTCGAATGCAGC |
| CX2 | CTAGATGCAGAAACCATAAG |
| CX3 | ATAGCAGTCATTAGCACTTC |
| CX8 | AGCTCTGCTGATGCCGTTGG |
| GA_30B_fwd | TTCGTGTTCTTGTCATTAATTAAATGCCTTATACAATCAACTCTCCG |
| GA_CP-PVX_rev | GGGTACCGGGCCCCCCCTCGAGGTCGACGGTATCGATAAGCTTGATATCGAATTCGCATGCAC |
| IKVAV-CP | ATCCGGCCGATGGCTGCATCTATTAAGGTTGCTGTTTCTGCTGATAGACCCGCGAGCACAACACAGCCCATAGGG |
| M13-uni | GTTGTAAAACGACGGCCAGT |
| M13-fw | GTT GTA AAA CGA CGG CCA GT |
| TMV5462f | TTGATGAGTTCATGGAAG |
| TMV6269r | TTCGATTTAAGTGGAGGG |
| TMV_RT_rev | ACGTGTGATTACGGACACAATC |
| TMV_seq_fwd | GAAGATGTCCCTATGTCGATCAG |
| NCAM1_fwd | AAGTGGCAGGAGATGC |
| NCAM1_rev | CAAA CTCGCCTGTAACCACACACT |
| NSE2_fwd | AGCTGGTGAAGGAAGCCATC |
| NSE2_rev | ATCGGGAAGGATCAGTGGGA |
| RBFOX3_fwd | CCTTGACTTCGGTCCTGGAG |
| RBFOX3_rev | GAGGTGAGGTCTGCTTTGCT |
| GAPDH_fwd | CAAGGTCATCCATGACAACTTTG |
| GAPDH_rev | GTCCACCACCCTGTTGCTGTAG |

**Section S1.** *Nucleotide sequence of recombinant viruses:*

The vectors pPVX-RGD, pPVX-IKVAV and pPVX-BDNF-2A are derived from pTCXIIc^[1]^ , while the vectors pTMV-RGD and pTMV-IKVAV are derived from pJL^[2]^. Upper case: sequences of recombinant virus *CP*, lower case: additional upstream sequences from *CP* subgenomic promotors^[3,4]^, black: coat protein, blue: functional target sequence, italics: FMDV 2A sequence.

pPVX-RGD

cgaaagaggtcagcaccagctagcatcgatccggccgATGGCTGGTAGAGGTGATAGCCCCGCGAGCACAACACAGCCCATAGGGTCAACTACCTCAACTACCACAAAAACTGCAGGCGCAACTCCTGCCACAGCTTCAGGCCTGTTCACCATCCCGGATGGGGATTTCTTTAGTACAGCCCGTGCCATAGTAGCCAGCAATGCTGTCGCAACAAATGAGGACCTCAGCAAGATTGAGGCTATTTGGAAGGACATGAAGGTGCCCACAGACACTATGGCACAGGCTGCTTGGGACTTAGTCAGACACTGTGCTGATGTAGGATCATCCGCTCAAACAGAAATGATAGATACAGGTCCCTATTCCAACGGCATCAGCAGAGCTAGACTGGCAGCAGCAATTAAAGAGGTGTGCACACTTAGGCAATTTTGCATGAAGTATGCTCCAGTGGTATGGAACTGGATGTTAACTAACAACAGTCCACCTGCTAACTGGCAAGCACAAGGTTTCAAGCCTGAGCACAAATTCGCTGCATTCGACTTCTTCAATGGAGTCACCAACCCAGCTGCCATCATGCCCAAAGAGGGGCTCATCCGGCCACCGTCTGAAGCTGAAATGAATGCTGCCCAAACTGCTGCCTTTGTGAAGATTACAAAGGCCAGGGCACAATCCAACGACTTTGCCAGCCTAGATGCAGCTGTCACTCGAGGTCGTATCACTGGAACAACAACCGCTGAGGCTGTTGTCACTCTACCACCACCA

pPVX-IKVAV

cgaaagaggtcagcaccagctagcatcgatccggccgATGGCTGCATCTATTAAGGTTGCTGTTTCTGCTGATAGACCCGCGAGCACAACACAGCCCATAGGGTCAACTACCTCAACTACCACAAAAACTGCAGGCGCAACTCCTGCCACAGCTTCAGGCCTGTTCACCATCCCGGATGGGGATTTCTTTAGTACAGCCCGTGCCATAGTAGCCAGCAATGCTGTCGCAACAAATGAGGACCTCAGCAAGATTGAGGCTATTTGGAAGGACATGAAGGTGCCCACAGACACTATGGCACAGGCTGCTTGGGACTTAGTCAGACACTGTGCTGATGTAGGATCATCCGCTCAAACAGAAATGATAGATACAGGTCCCTATTCCAACGGCATCAGCAGAGCTAGACTGGCAGCAGCAATTAAAGAGGTGTGCACACTTAGGCAATTTTGCATGAAGTATGCTCCAGTGGTATGGAACTGGATGTTAACTAACAACAGTCCACCTGCTAACTGGCAAGCACAAGGTTTCAAGCCTGAGCACAAATTCGCTGCATTCGACTTCTTCAATGGAGTCACCAACCCAGCTGCCATCATGCCCAAAGAGGGGCTCATCCGGCCACCGTCTGAAGCTGAAATGAATGCTGCCCAAACTGCTGCCTTTGTGAAGATTACAAAGGCCAGGGCACAATCCAACGACTTTGCCAGCCTAGATGCAGCTGTCACTCGAGGTCGTATCACTGGAACAACAACCGCTGAGGCTGTTGTCACTCTACCACCACCA

pPVX-BDNF-2A

cgaaagaggtcagcaccagctagcgctaccggtcgccaccATGCGAGGCATCGACAAACGTCATTGGAACTCACAAGGAGGC*TCCGGATCTAGAAATTTTGACCTTCTTAAGCTTGCGGGAGACGTCGAGTCCAACCCCGGG*CCCGCGAGCACAACACAGCCCATAGGGTCAACTACCTCAACTACCACAAAAACTGCAGGCGCAACTCCTGCCACAGCTTCAGGCCTGTTCACTATCCCGGATGGGGATTTCTTTAGTACAGCCCGTGCCATAGTAGCCAGCAATGCTGTCGCAACAAATGAGGACCTCAGCAAGATTGAGGCTATTTGGAAGGACATGAAGGTGCCCACAGACACTATGGCACAGGCTGCTTGGGACTTAGTCAGACACTGTGCTGATGTAGGATCATCCGCTCAAACAGAAATGATAGATACAGGTCCCTATTCCAACGGCATCAGCAGAGCTAGACTGGCAGCAGCAATTAAAGAGGTGTGCACACTTAGGCAATTTTGCATGAAGTATGCCCCAGTGGTATGGAACTGGATGTTAACTAACAACAGTCCACCTGCTAACTGGCAAGCACAAGGTTTCAAGCCTGAGCACAAATTCGCTGCATTCGACTTCTTCAATGGAGTCACCAACCCAGCTGCCATCATGCCCAAAGAGGGGCTCATCCGGCCACCGTCTGAAGCTGAAATGAATGCTGCCCAAACTGCTGCCTTTGTGAAGATTACAAAGGCCAGGGCACAATCCAACGACTTTGCCAGCCTAGATGCAGCTGTCACTCGAGGTCGTATCACTGGAACAACAACCGCTGAGGCTGTTGTCACTCTACCACCACCA

pTMV-RGD

gatcttacagtatcactactccatctcagttcgtgttcttgtcattaattaaATGCCTTATACAATCAACTCTCCGAGCCAATTTGTTTACTTAAGTTCCGCTTATGCAGATCCTGTGCAGCTGATCAATCTGTGTACAAATGCATTGGGTAACCAGTTTCAAACGCAACAAGCTAGGACAACAGTCCAACAGCAATTTGCGGATGCCTGGAAACCTGTGCCTAGTATGACAGTGAGATTTCCTGCATCGGATTTCTATGTGTATAGATATAATTCGACGCTTGATCCGTTGATCACGGCGTTATTAAATAGCTTCGATACTAGAAATAGAATAATAGAGGTTGATAATCAACCCGCACCGAATACTACTGAAATCGTTAACGCGACTCAGAGGGCAGACGATGCGACTGTAGCTATAAGGGCTTCAATCAATAATTTGGCTAATGAACTGGTTCGTGGAACTGGCATGTTCAATCAAGCAAGCTTTGAGACTGCTAGTGGACTTGTCTGGACCACAACTCCGGCTACTGCTGGTAGAGGTGATAGC

pTMV-IKVAV

gatcttacagtatcactactccatctcagttcgtgttcttgtcattaattaaATGCCTTATACAATCAACTCTCCGAGCCAATTTGTTTACTTAAGTTCCGCTTATGCAGATCCTGTGCAGCTGATCAATCTGTGTACAAATGCATTGGGTAACCAGTTTCAAACGCAACAAGCTAGGACAACAGTCCAACAGCAATTTGCGGATGCCTGGAAACCTGTGCCTAGTATGACAGTGAGATTTCCTGCATCGGATTTCTATGTGTATAGATATAATTCGACGCTTGATCCGTTGATCACGGCGTTATTAAATAGCTTCGATACTAGAAATAGAATAATAGAGGTTGATAATCAACCCGCACCGAATACTACTGAAATCGTTAACGCGACTCAGAGGGCAGACGATGCGACTGTAGCTATAAGGGCTTCAATCAATAATTTGGCTAATGAACTGGTTCGTGGAACTGGCATGTTCAATCAAGCAAGCTTTGAGACTGCTAGTGGACTTGTCTGGACCACAACTCCGGCTACTGCTGCATCTATTAAGGTTGCTGTTTCTGCTGATAGA

**Table S2.** Reagent volumes for the MasterMix for qPCR. Displayed volumes were multiplied by the number of samples.

| **Solution** | **Volume** |
| --- | --- |
| SYBR Green PCR MasterMix | 5 µL |
| Forward primer of the subsequent gene | 0.5 µL |
| Reverse primer of the subsequent gene | 0.5 µL |
| RNase-free water | 3 µL |

**Table S3.** Antibody dilutions for immunostaining.

| **Antibody** | **Dilution Factor in 0.2% BSA in PBS** | **Incubation Conditions** |
| --- | --- | --- |
| Mouse anti-β3-tubulin (Santa Cruz Biotechnology, Dallas, USA) | 1:100 | overnight at 4 °C |
| Rabbit anti-PVX CP (DSMZ, Braunschweig, Germany) | 1:400 | overnight at 4 °C |
| Rabbit anti-TMGMV CP (DSMZ, Braunschweig, Germany) | 1:400 | Overnight at 4 °C |
| Anti-mouse Alexa 555 (Invitrogen by Thermo Fisher Scientific) | 1:2000 | Two hours at room temperature |
| Anti-rabbit Alexa 488 (Invitrogen by Thermo Fisher Scientific) | 1:2000 | Two hours at room temperature |

**Section S2.** *Confirmation of FAK isomers expression:* SH-SY5Y cells were seeded on the 12-well plates and incubated overnight, so that confluency above 90% was reached. The next day, cells were exposed to PVX-RGD or PVX-IKVAV peptides for 1 hour, at the concentration of 50 µg/mL, in 500 µL volume per well. After 1 hour, samples were washed with PBS and lysed for further RNA isolation, cDNA synthesis and qPCR, performed as previously described in this paper. Primers for FAK isomers were designed with primer BLAST.

**Section S3.** *Immunoprecipitation – Isolation of the complex from µColumns:* For the immunoprecipitation, µColumns (Miltenyi Biotec) were attached to a stand magnet (Miltenyi Biotec). The column was activated by rinsing with 200 µL of IP lysis buffer (5 mM MgCl_2_, 137 mM KCl, 1 mM EDTA, 1 mM EGTA, 1% CHAPSO (Sigma Aldrich Chemie, St. Louis, USA), and 20 mM Tris HCl pH7.5 (Tris Hydrochloride MB, Merck Chemicals, Gernsheim). Then, the microbead-antibody-receptor complex was pipetted into the column followed by washing the column three times with 1 mL of IP lysis buffer and finally with 0.5 mL of low wash buffer (20 mM Tris HCl pH 7.5). After the low wash buffer completely ran through, elution buffer (1X Laemmli (4X Laemmli, BioRad, Hercules, USA), 50 mM DTT) was added at a volume of 20 µL directly into the middle of the column. During an incubation time of 5 minutes, droplets potentially occurring at the outlet of the microcolumn were removed. Next, a 1.5 mL tube (Eppendorf, Hamburg) was put under the outlet of the column and 50 µL elution buffer was added to the column, the flow-through contained the proteins of interest, and the primary antibody.


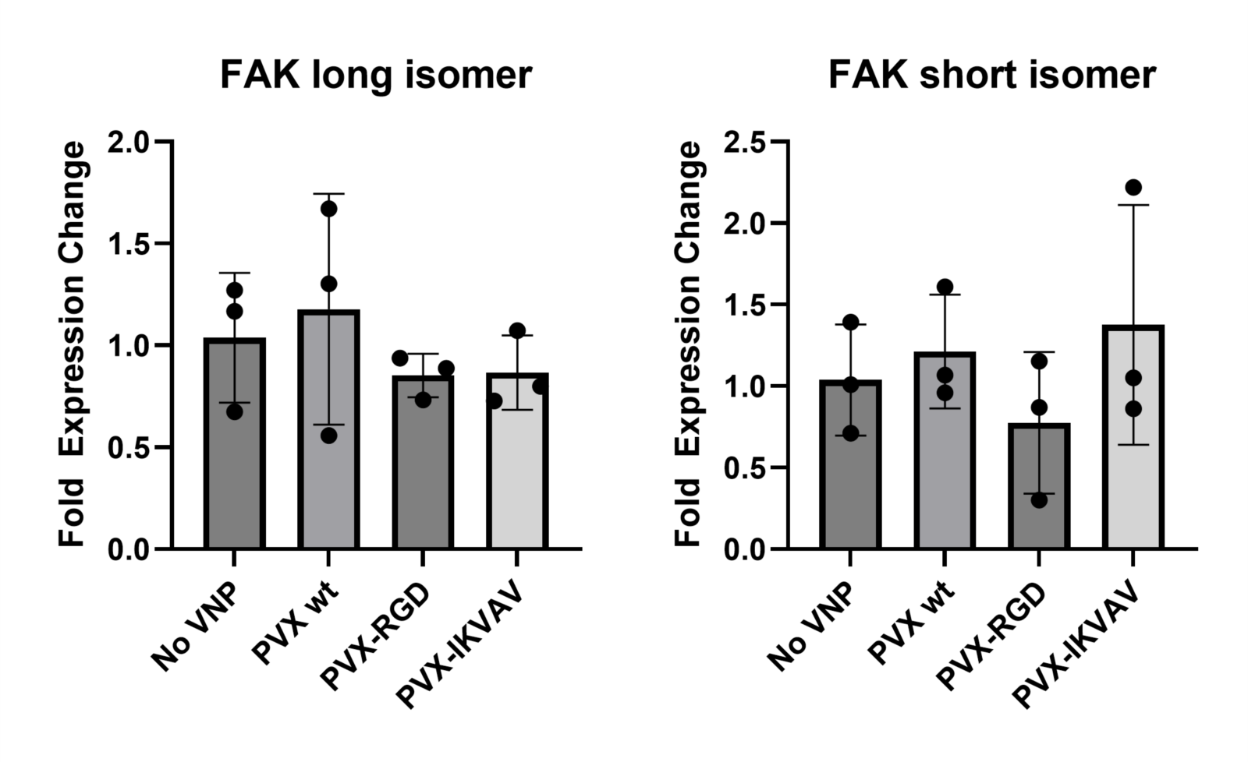
**Figure S1.** Expression levels between different Focal Adhesion Kinase Isomers. Both tested forms are expressed. Long isomer codes for the 140kDa protein, while short isomer codes for 70 kDa isomer. Both isomers were tested in cells cultivated without VNPs, PVX wt, PVX-RGD and PVX-IKVAV; n = 3.


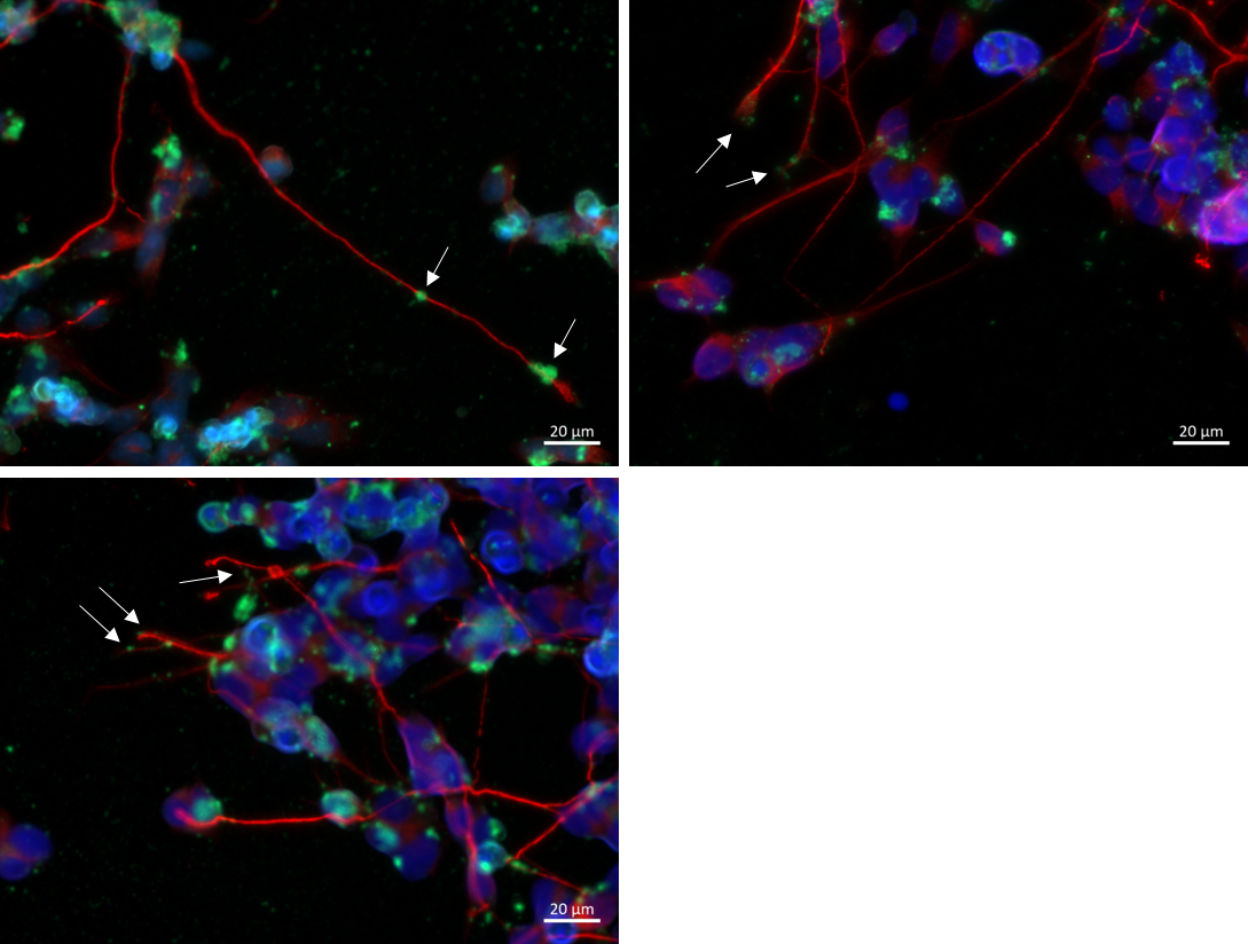


**Figure S2.** Interactions of PVX-BDNF-2A VNPs alongside neurites. Red color represents the neuronal marker β3-tubulin, cell nuclei are stained blue and green represents the PVX-BDNF-2A particles. Scale bar is 20 µm.


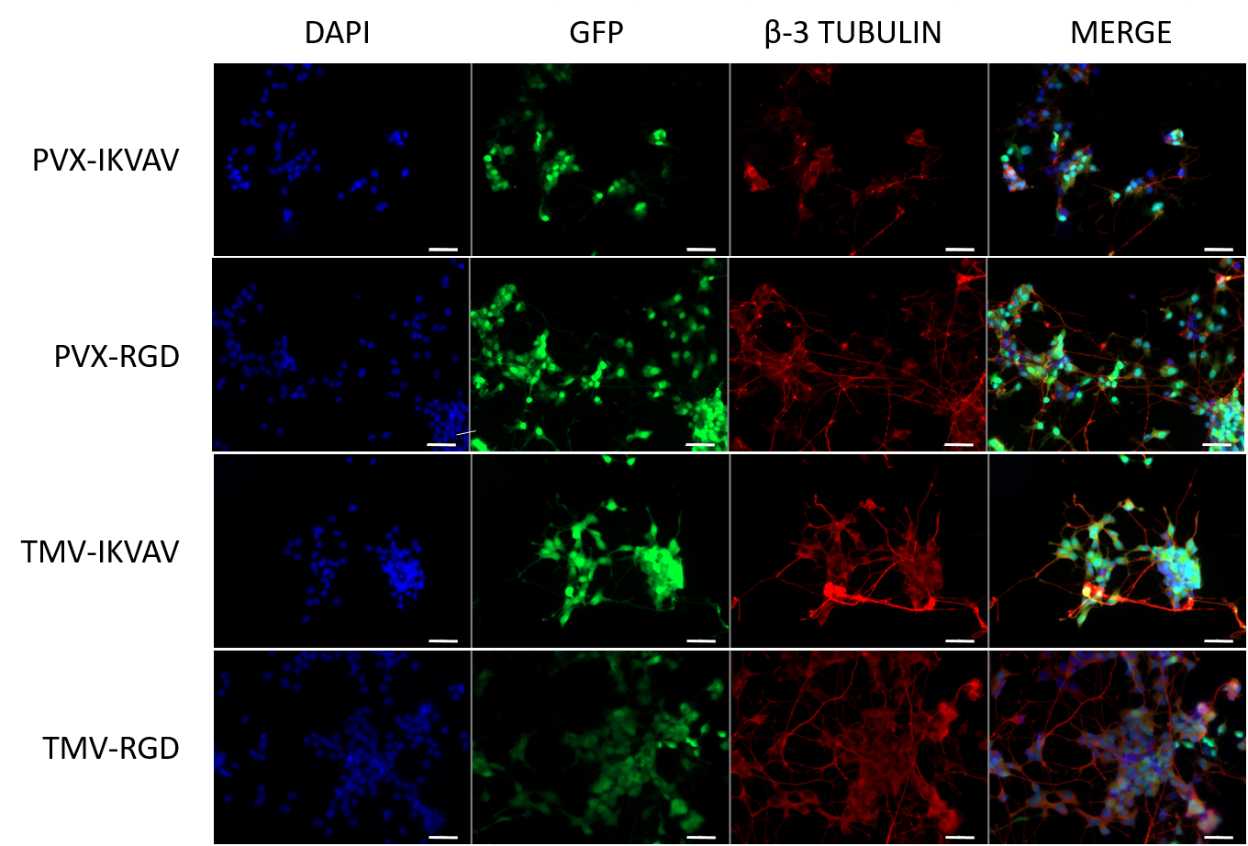


**Figure S3.** Expression of the neuronal marker β3-tubulin by neuroblastoma cells differentiated with the VNPs PVX-IKVAV, PVX-RGD, TMV-IKVAV or TMV-RGD. Nuclei are blue, cell bodies are green and the neuronal marker β3-tubulin is red. Scale bar represents 50 µm.


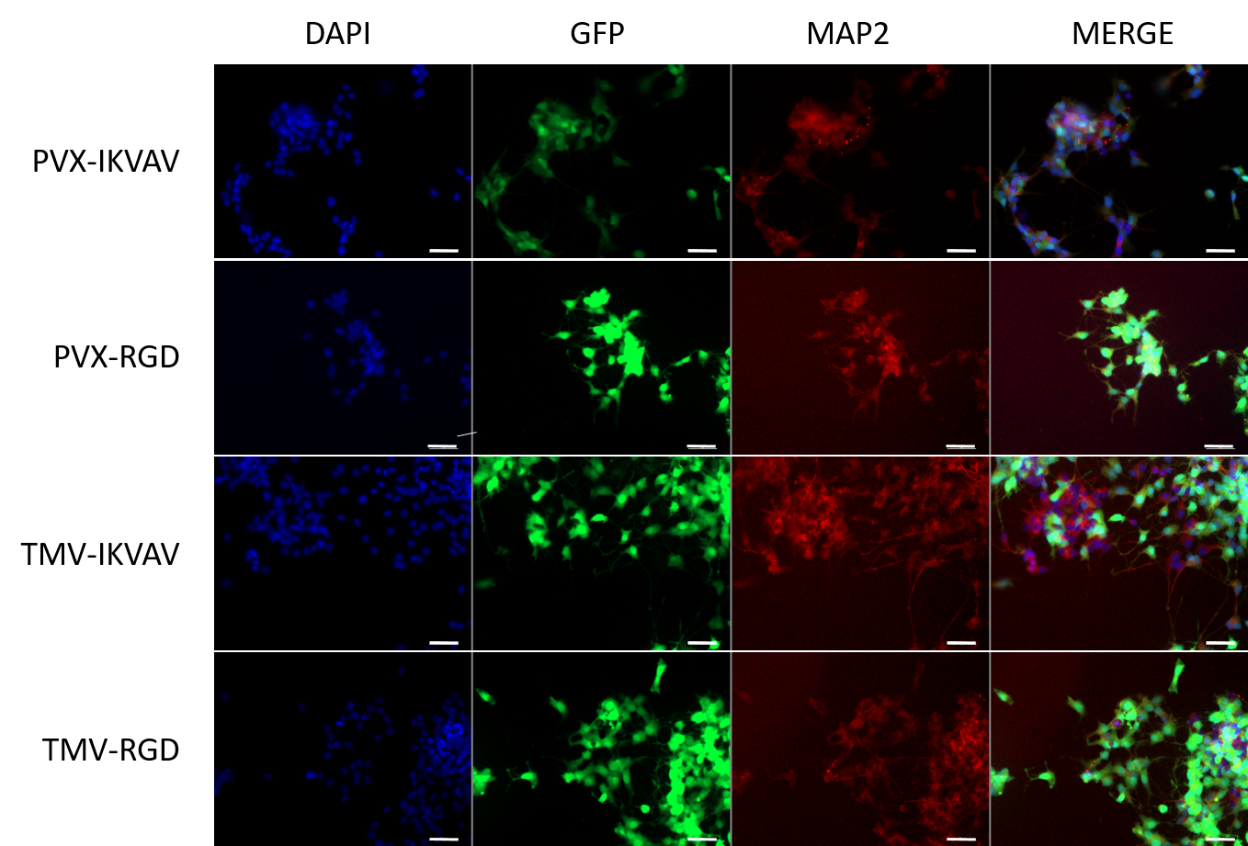


**Figure S4.** Expression of the neuronal marker MAP2 by neuroblastoma cells differentiated with the VNPs PVX-IKVAV, PVX-RGD, TMV-IKVAV or TMV-RGD. Nuclei are blue, cell bodies are green and the neuronal marker MAP2 is red. Scale bar represents 50 µm.


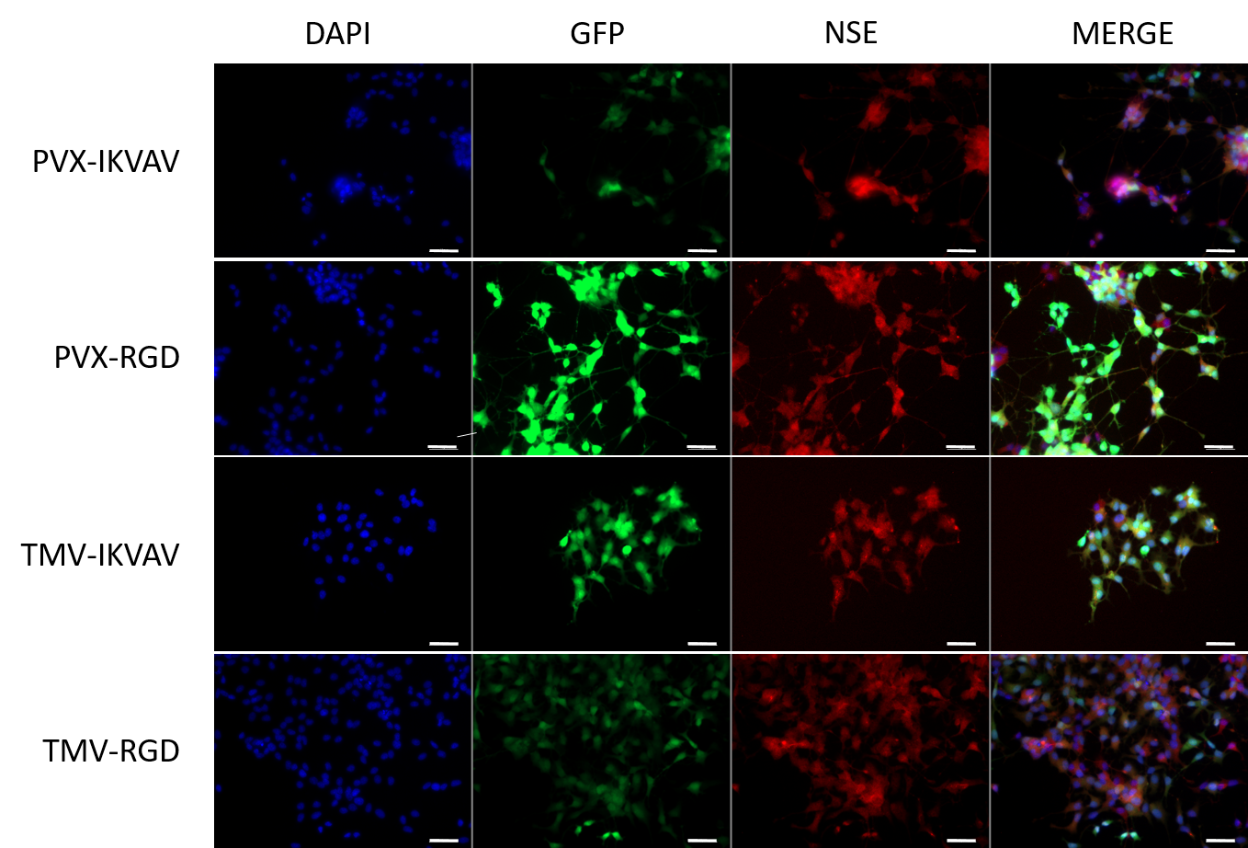


**Figure S5.** Expression of the neuronal marker NSE by neuroblastoma cells differentiated with the VNPs PVX-IKVAV, PVX-RGD, TMV-IKVAV or TMV-RGD. Nuclei are blue, cell bodies are green and the neuronal marker NSE is red. Scale bar represents 50 µm.


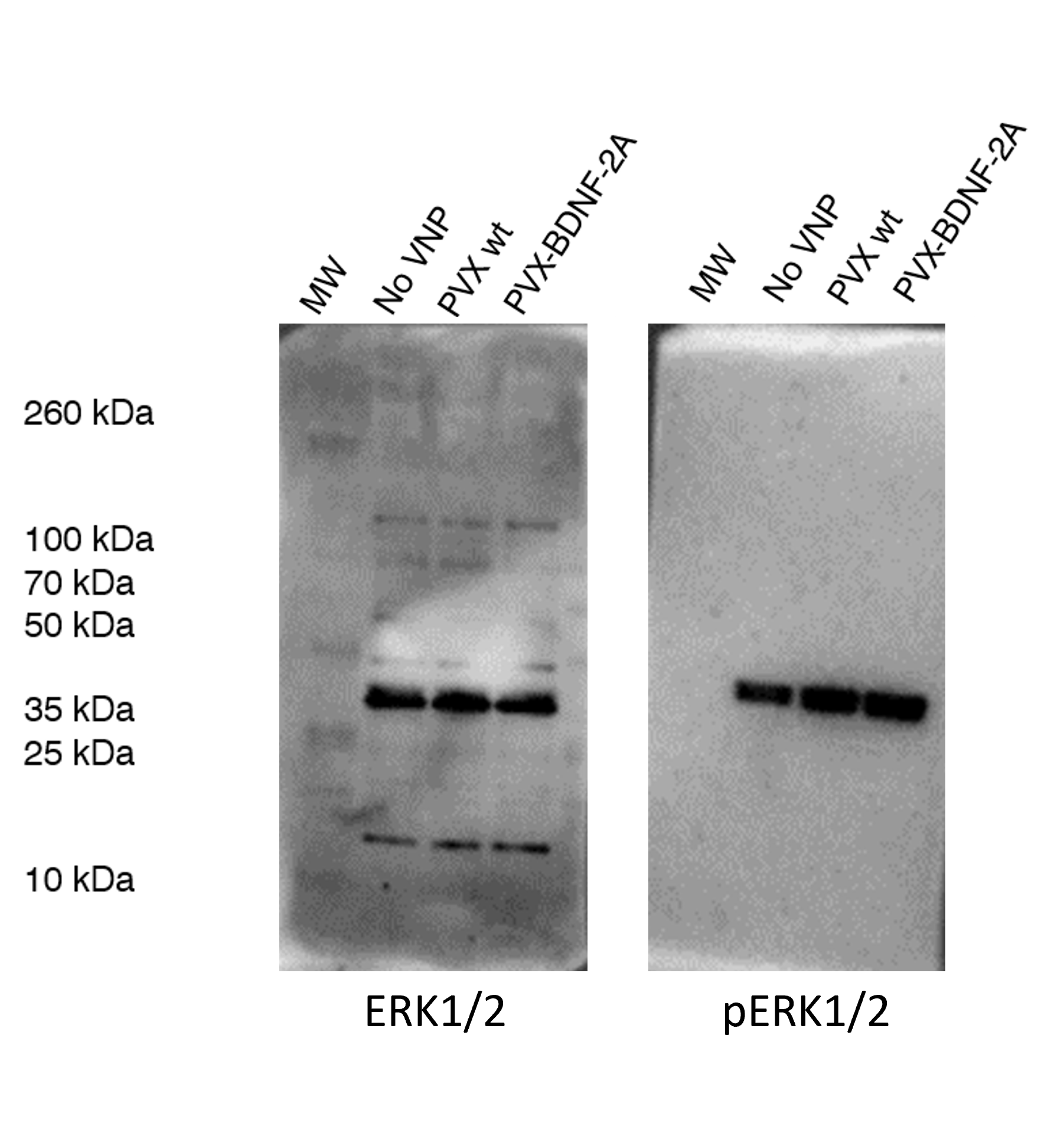


**Figure S6.** Intracellular pathway activation following to PVX-BDNF-2A binding to the tyrosine-receptor. The relation of p44/42 MAPK (ERK1/2) and its phosphorylated state was investigated. Cells incubated with no VNPs and PVX wt were used as a control. Western Blot membranes were stained with anti-ERK1/2 (Santa Cruz, Dallas, USA) or anti-pERK1/2 (cell signaling technology, Danvers, USA), respectively.


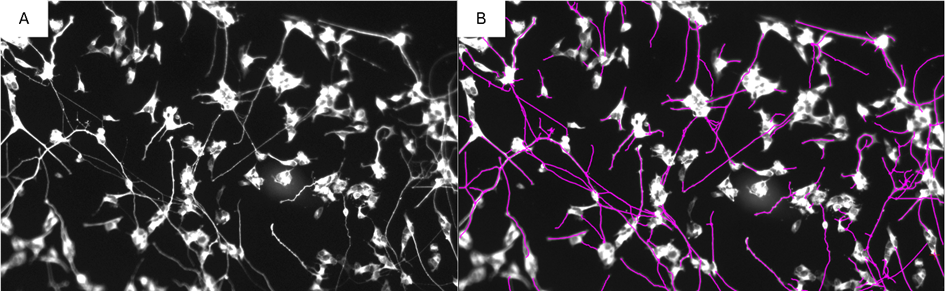


**Figure S7.** Example for the tracings made with SNT plugin of the Fiji software to analyze the characteristics of the neurites. A: Immunofluorescence image of the channel stained for β3 tubulin of differentiated neuroblastoma cells. Cells visible in the image were counted. B: Immunofluorescence overlayed with the tracings added by hand using the SNT plugin of the Fiji app. Traces were used to analyze the length and number of neurites and number of branches visible in one image related to the number of cells visible.


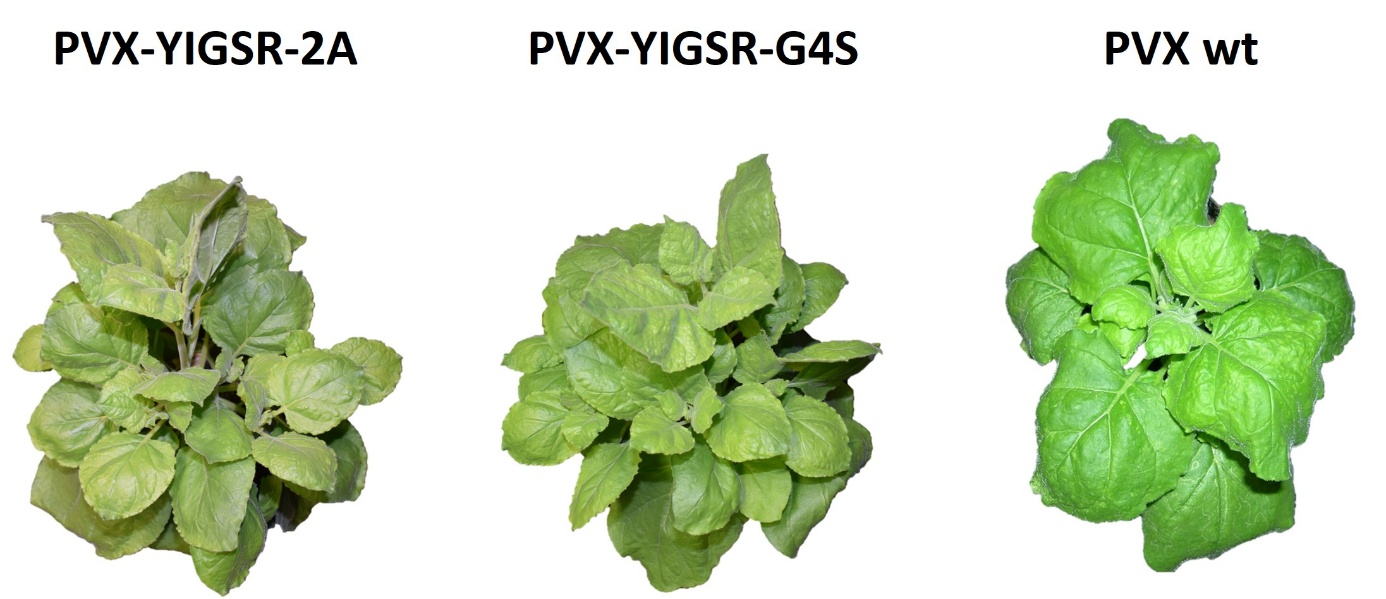


**Figure S8.** Absence of systemic infection at 20 dpi in N. benthamiana plants inoculated with PVX vectors designed for the production of PVX-YIGSR nanoparticles. The YIGSR sequence was fused to the 5′ end of PVX coat protein ether via an additional FMDV 2A ribosomal skipping sequence or through a flexible glycine-serine rich linker (G4S). In contrast to infection with PVX wild-type (wt) particles, which show typical systemic infection symptoms such as dwarfing and leaf crinkling (15 dpi), neither fusion construct resulted in systemic spread of the VNP.

**References**

1. Shukla S, Dickmeis C, Nagarajan AS, Fischer R, Commandeur U, Steinmetz NF. Molecular farming of fluorescent virus-based nanoparticles for optical imaging in plants, human cells and mouse models. *Biomater Sci,* 784–797 (2014).
2. Lindbo JA. TRBO: A high-efficiency tobacco mosaic virus RNA-based overexpression vector. *Plant Physiol* 145, 1232-1240, [10.1104/pp.107.106377](https://doi.org/10.1104/pp.107.106377) (2007).
3. Goelet P, Lomonssoff GP, Akam ME, Gait MJ, Karn J. Nucleotide sequence of tobacco mosaic virus RNA. *Proc Natl Acad Sci U S A* 79**,** 5818-5822, 10.1073/pnas.79.19.5818 (1982).
4. Kim KH, Hemenway CL. Long-distance RNA-RNA interactions and conserved sequence elements affect potato virus X plus-strand RNA accumulation. *RNA*, 636-645 (1999).
